# From Agri-Food Byproducts to High-Value Bioactive Compounds: A Critical Review Linking Green Recovery and Chemical Profiling to Circular Valorization

**DOI:** 10.3390/molecules31122136

**Published:** 2026-06-17

**Authors:** Hyo Jun Won, Ae-jin Choi

**Affiliations:** Food Tech Resources Research Division, National Institute of Crop and Food Science (NICS), Rural Development Administration (RDA), Wanju-gun 55365, Republic of Korea; hjwon7943@ust.ac.kr

**Keywords:** agri-food byproducts, bioactive compounds, circular economy, green recovery, chemical profiling, metabolomics, bioactivity evaluation, quality markers, standardization, application readiness

## Abstract

Agri-food byproducts are increasingly recognized as sustainable feedstocks for high-value bioactive compounds; but their practical valorization requires integrated evidence on recovery conditions; chemical composition; bioactivity; and application readiness. This review critically examines green recovery strategies and chemical profiling platforms for bioactive compounds recovered from peels; pomace; seed residues; hulls; vegetation waters; and pruning waste. Emphasis is placed on how extraction variables shape chemical profiles; extract quality; and reported biological activities. Ultrasound- and microwave-assisted extraction; enzyme- and fermentation-assisted recovery; supercritical fluid extraction; pressurized liquid extraction; pulsed electric field-assisted pretreatment; and green solvent-based extraction are discussed in terms of target-compound selectivity; solvent and energy demand; process safety; scalability; and sustainability-related evidence. Chromatographic; mass-spectrometric; spectroscopic; and metabolomics-based profiling approaches are evaluated for identification; annotation; quantification; fingerprinting; quality-marker selection; and standardization; with confidence levels distinguished according to authentic-standard matching; tandem mass spectrometry evidence; spectral libraries; or fingerprint-level evidence. Circular valorization pathways in food; nutraceutical; cosmetic; pharmaceutical, and biopesticide-related applications are further considered with attention to feedstock heterogeneity; process standardization; stability; safety; regulatory feasibility; scalability; and techno-economic feasibility. Overall; this review provides a linkage-oriented framework for developing standardized; application-readiness-oriented bioactive candidates from agri-food byproducts.

## 1. Introduction

Agri-food systems generate substantial residual biomass and liquid or semi-liquid byproduct streams during harvesting, postharvest handling and storage, primary processing, and industrial transformation. These streams—including peels, pomace, seed residues, hulls and bran fractions, stems, leaves, pruning waste, pressing residues, and vegetation waters—are often discarded, underused, or directed to low-value applications despite their potential as chemically rich sources of recoverable bioactive compounds [1,2,3]. Within circular economy and biorefinery frameworks, these byproduct streams are increasingly being reconsidered as renewable matrices for recovering high-value bioactive compounds [2,4,5]. These compounds include phenolics, flavonoids, carotenoids, tocopherols, terpenoids, peptides, polysaccharides, dietary fibers, fatty acids, and other recoverable constituents, although their distribution and recoverability depend on matrix type and processing history [2,3,5]. In this review, “bioactive compounds” are operationally defined as chemically identifiable molecules or chemically characterized fractions recovered from plant-derived agri-food byproducts that are associated with biological or biologically relevant activity under defined assay conditions or application contexts. This definition distinguishes bioactivity from nutritional value, which primarily refers to nutrient supply, digestibility, or energy-related contribution, and from technological functionality, which refers to physicochemical performance such as thickening, gelation, emulsification, stabilization, color, aroma, water binding, or carrier behavior. Accordingly, this review frames agri-food byproduct valorization as a shift from waste management toward chemically guided resource recovery, where biomass composition, extraction selectivity, analytical characterization, and application requirements jointly determine valorization potential.

The valorization potential of agri-food byproducts arises from matrix-dependent chemical profiles rather than from chemical richness alone [1,2]. Botanical origin, tissue type, maturity, processing history, and storage conditions can influence the abundance, accessibility, and stability of recoverable functional fractions, including polyphenols (phenolic acids, flavonoids, tannins, anthocyanins, and stilbenes), lipophilic constituents (carotenoids, tocopherols, and fatty acids), volatile and semi-volatile terpenoids, essential-oil constituents, and macromolecular or structurally associated fractions such as dietary fibers, peptides, and polysaccharides [1,2,3]. Because these fractions differ in polarity, molecular size, volatility, matrix binding, and degradation behavior, valorization requires profile-guided recovery and chemical characterization rather than a simple inventory of bioactive constituents [2,4,6]. Reported antioxidant, antimicrobial, anti-inflammatory, cytoprotective, photoprotective, and enzyme-modulatory activities have supported interest in food, nutraceutical, cosmetic, and pharmaceutical applications [2,3,7]. Plant-protective activities further support biopesticide-related and agricultural applications [8]. However, such functional potential becomes application-relevant only when the recovered extract has a defined chemical identity, reproducible quality attributes, demonstrated stability, an acceptable safety profile, formulation compatibility, scalability, regulatory feasibility, and sustainability performance [2,9,10,11].

Green recovery strategies are central to agri-food byproduct valorization because the recovery step determines not only extraction efficiency but also compound selectivity, chemical integrity, co-extracted matrix components, and downstream compatibility [2,12]. Ultrasound-assisted, microwave-assisted, enzyme-assisted, supercritical fluid, pressurized liquid, and pulsed electric field-assisted extraction have been explored to recover chemically diverse bioactives while reducing solvent use, processing time, energy demand, and environmental burden [2,3,9]. Fermentation-assisted recovery and green solvent- or DES/NADES-based extraction further expand this toolbox by enabling biologically mediated release, biotransformation, and solvent-system design for matrix- and compound-specific recovery [13,14]. However, these methods are matrix- and compound-class-specific rather than universally interchangeable: solvent polarity, mass-transfer mechanism, temperature or pressure regime, and process intensity influence whether a process preferentially recovers polar phenolics, lipophilic carotenoids, tocopherols, fatty acids, volatile terpenoids, or other target fractions [2,3,4,9]. Enzymatic and fermentation-assisted strategies add a further layer of specificity because they can release, hydrolyze, or transform glycosylated, polymerized, protein-associated, or cell-wall-bound metabolites [13,15,16]. Therefore, green recovery should be evaluated through an integrated set of chemical and translational criteria—including selectivity, chemical stability, extract identity, solvent and process safety, regulatory acceptability, scalability, and application-specific compatibility—rather than by extraction yield alone [2,9,10,11].

Chemical profiling provides the evidence needed to define heterogeneous byproduct-derived extracts as chemically interpretable materials that can be compared, standardized, and evaluated for application-specific use [2,6]. Chromatographic, mass-spectrometric, spectroscopic, and metabolomics-based workflows provide complementary layers of evidence for compound identification, relative or absolute quantification, fingerprint comparison, quality-marker selection, and batch-level standardization [2,4,6]. This step is particularly important for agri-food byproduct extracts, which may contain structurally related metabolites, co-extracted matrix components, degradation products, and constituents that interact synergistically or antagonistically [2,4,17]. Thus, in this review, profiling is treated not as a post-extraction description but as an interpretive layer that links recovery conditions to extract identity, reproducibility, and bioactivity interpretation. Without sufficient characterization, reported biological responses remain difficult to compare across studies, reproduce across batches, or translate into sector-specific applications [2,6].

Recent reviews have provided valuable overviews of agri-food waste streams, major bioactive compound classes, green recovery technologies, analytical characterization methods, and application routes within circular bioeconomy frameworks [1,2,4]. Collectively, these reviews establish agri-food byproducts as important reservoirs of functional molecules; however, their primary organizing focus is not always the mechanistic and translational linkage among recovery conditions, chemical profiles, extract quality, bioactivity interpretation, and application readiness. In particular, extraction variables are often evaluated through yield, total phenolic content, total flavonoid content, or preliminary bioactivity metrics without being consistently traced to compound-level identity, marker reproducibility, chemical stability, co-extracted constituents, and sector-specific performance requirements [2,6,18]. This gap limits cross-study comparability and may obscure whether a chemically active extract is also standardized, safe, scalable, and suitable for a defined circular valorization pathway. A more chemistry-centered synthesis is therefore needed to connect matrix selection and green recovery with analytical profiling, bioactivity evidence, and application-specific readiness criteria.

To address this need, this review critically examines plant-derived agri-food byproducts as chemically heterogeneous and sustainable feedstocks for high-value bioactive compounds. Building on the recent reviews discussed above, it adopts a linkage-oriented framework that traces how matrix properties and green recovery conditions shape chemical profiles, extract quality, bioactivity interpretation, and application readiness. By integrating recovery selectivity, fit-for-purpose chemical profiling, marker-based standardization, stability and safety considerations, and sector-specific translational bottlenecks, this review aims to provide a chemistry-centered basis for converting agri-food byproducts into standardized, application-readiness-oriented bioactive extracts and fractions within circular valorization pathways.

## 2. Review Scope and Literature Collection Strategy

### 2.1. Review Design and Conceptual Scope

This article was designed as a critical narrative review rather than a systematic review, scoping review, quantitative meta-analysis, or bibliometric study. The literature collection strategy was used to make source identification and selection transparent, but the primary purpose of the review was interpretive rather than enumerative. Accordingly, formal PRISMA-style record enumeration, flow-diagram reporting, study-level risk-of-bias scoring, bibliometric mapping, and quantitative synthesis were not applied. Instead, evidence was prioritized according to its relevance to the matrix–recovery–profile–bioactivity–application-readiness linkage framework. Specifically, this review synthesizes how matrix characteristics, green recovery conditions, chemical profiles, bioactivity evidence, and application-readiness criteria are connected. Therefore, studies were selected not merely to catalogue all publications in the field, but to clarify how agri-food byproducts can be transformed into chemically defined, standardized, and application-specific bioactive extracts and fractions.

The conceptual scope primarily covers plant-derived agri-food byproduct streams, including both solid residues and liquid or semi-liquid processing streams, generated during harvesting, postharvest handling, food processing, and industrial transformation. Representative matrices include peels, pomace, seed residues, hulls, shells, bran fractions, pressing residues, vegetation waters, leaves, stems, and pruning waste. The review emphasizes bioactive compounds and chemically characterized recoverable fractions commonly obtained from these matrices, including polyphenols such as phenolic acids, flavonoids, tannins, anthocyanins, and stilbenes; lipophilic constituents such as carotenoids, tocopherols, and fatty acids; volatile or semi-volatile constituents such as terpenoids and essential oil constituents; and macromolecular or structurally associated fractions such as dietary fibers, peptides, and polysaccharides. Particular attention is given to studies that explicitly link byproduct matrices and recovery conditions with chemical profiling, bioactivity interpretation, extract quality, and sector-specific valorization in food, nutraceutical, cosmetic, pharmaceutical, biopesticide-related, and agricultural contexts.

For conceptual consistency, the term “bioactive compounds” is used here as an operational category rather than as a purely compositional label. The term refers to chemically identifiable molecules or chemically characterized fractions whose reported relevance depends on biological or biologically related endpoints, such as antioxidant, antimicrobial, anti-inflammatory, cytoprotective, photoprotective, enzyme-modulatory, plant-protective, or other sector-relevant responses. By contrast, nutritional value refers to nutrient supply, digestibility, energy contribution, or macro- and micronutrient relevance, whereas technological functionality refers to material performance in a product matrix, including water binding, viscosity modification, gelation, emulsification, stabilization, color or aroma contribution, encapsulation, and carrier behavior. Nutritional and technological properties are considered important application-readiness attributes in this review, but they are not treated as evidence of bioactivity unless they are linked to a defined chemical profile and a relevant biological or application-specific endpoint.

### 2.2. Literature Sources and Search Strategy

As this was a critical narrative review, the literature was identified through iterative and targeted keyword searches in Scopus, Web of Science, and PubMed, complemented by Google Scholar and backward and forward citation chasing. The strategy was intended to support transparent source selection rather than exhaustive systematic retrieval or quantitative screening. Scopus, Web of Science, and PubMed were used as the primary databases, whereas Google Scholar was used as a supplementary tool for citation chasing, identifying additional highly relevant records, and cross-checking references cited in key review papers. The search prioritized peer-reviewed original articles and review papers published in indexed journals, with emphasis on recent studies and highly relevant foundational papers related to green recovery, chemical profiling, bioactivity interpretation, safety, sustainability assessment, and circular valorization. No strict publication-year restriction was imposed; however, priority was given to recent literature, particularly studies published from 2021 through May 2026, together with foundational methodological papers where relevant. Search strings were adapted to each database and combined keywords using Boolean operators and truncation where supported. The final literature search update was conducted in May 2026 before manuscript submission.

The search strategy was organized around six conceptual blocks: byproduct matrices, target compound classes, green recovery technologies, analytical profiling platforms, biological activities, and valorization or application-readiness criteria. Representative matrix-related terms included “agri-food byproducts”, “agri-food waste”, “agro-industrial byproducts”, “food processing byproducts”, “peels”, “pomace”, “seed residues”, “hulls”, “shells”, “bran”, “pressing residues”, “vegetation waters”, “olive mill wastewater”, “aqueous effluents”, “leaves”, “stems”, and “pruning waste”. Target-compound terms included “bioactive compounds”, “phenolic compounds”, “polyphenols”, “flavonoids”, “anthocyanins”, “tannins”, “stilbenes”, “carotenoids”, “tocopherols”, “fatty acids”, “phytosterols”, “terpenoids”, “essential oils”, “secoiridoids”, “limonoids”, “peptides”, “polysaccharides”, “pectin”, “dietary fiber”, and “bound phenolics”. Recovery-related terms included “green extraction”, “green recovery”, “ultrasound-assisted extraction”, “microwave-assisted extraction”, “enzyme-assisted extraction”, “fermentation-assisted recovery”, “supercritical fluid extraction”, “supercritical CO_2_”, “pressurized liquid extraction”, “pressurized hot-water extraction”, “subcritical water extraction”, “pulsed electric field-assisted extraction”, “hydrodistillation”, “steam distillation”, “solvent-free microwave extraction”, “deep eutectic solvents”, “natural deep eutectic solvents”, “food-grade solvents”, “GRAS solvents”, and “bio-based solvents”. Analytical and interpretation terms included “chemical profiling”, “phytochemical profiling”, “HPLC”, “HPLC-DAD”, “UHPLC”, “UHPLC-DAD”, “LC-MS”, “LC-MS/MS”, “LC-HRMS”, “GC-MS”, “GC-FID”, “NMR”, “FTIR”, “NIR”, “Raman”, “metabolomics”, “fingerprinting”, “chemometrics”, “quality markers”, “standardization”, “annotation confidence”, “retention index”, “negative markers”, “contaminants”, and “residual solvents”. Additional combinations included “bioactivity”, “antioxidant”, “antimicrobial”, “antifungal”, “anti-inflammatory”, “cytoprotective”, “photoprotective”, “enzyme-modulatory”, “plant-protective”, “circular economy”, “circular valorization”, “biorefinery”, “functional food”, “nutraceuticals”, “supplements”, “cosmetics”, “pharmaceutical-oriented discovery”, “biopesticides”, “sustainable agriculture”, “bioaccessibility”, “bioavailability”, “stability”, “safety”, “formulation compatibility”, “scale-up”, “regulatory feasibility”, “techno-economic analysis”, and “life cycle assessment”.

### 2.3. Relevance Criteria and Scope Boundaries

Studies were considered within the core scope when they addressed at least one major component of the review framework and provided sufficient methodological, chemical, biological, or translational information relevant to agri-food byproduct-derived bioactive compounds. Core studies included those that investigated the recovery of bioactive compounds from defined plant-derived agri-food byproduct matrices or streams; compared conventional and green recovery approaches; characterized byproduct-derived extracts using chromatographic, mass-spectrometric, spectroscopic, fingerprinting, or metabolomics-based platforms; linked extraction or pretreatment variables to chemical composition, extract quality, or marker profiles; evaluated biological activities in relation to food, nutraceutical, cosmetic, pharmaceutical, biopesticide-related, or agricultural applications; or discussed standardization, safety, regulatory feasibility, scale-up, techno-economic feasibility, sustainability assessment, or circular valorization of byproduct-derived bioactive resources.

Studies were considered outside the main scope when they focused exclusively on energy production, composting, anaerobic digestion, animal feed, waste disposal, municipal solid waste, mixed household food waste, or animal-derived byproducts without a clearly defined connection to plant-derived agri-food byproduct matrices or streams and recoverable bioactive compounds. Studies on treatment, detoxification, or reuse of byproduct streams were retained only when they were explicitly linked to recoverable bioactive compounds, chemical profiling, safety, application readiness, or circular valorization. Publications reporting only extraction yield, total phenolic content, total flavonoid content, or single bulk antioxidant-capacity measurements without sufficient information on the byproduct source, recovery conditions, analytical characterization, or chemical composition were not used as core evidence. Such studies were considered only when they provided useful contextual information for methodological comparison, historical background, or application-oriented discussion.

### 2.4. Critical Synthesis Framework

The literature considered in this review was synthesized narratively using a linkage-oriented framework organized around five analytical dimensions: (i) byproduct matrix and target chemistry, (ii) green recovery design, (iii) chemical profiling and standardization, (iv) profile-linked bioactivity interpretation, and (v) application readiness and translational constraints. No quantitative meta-analysis, study-level risk-of-bias assessment, or bibliometric mapping was performed. Byproduct matrices were first grouped according to botanical or processing origin, tissue structure, physical state, and dominant compound classes. Green recovery strategies were then compared in terms of extraction mechanism, selectivity, process efficiency, solvent compatibility, energy demand, compound stability, and scale-up potential. Chemical profiling platforms were evaluated for their ability to support compound identification or annotation, quantification, fingerprinting, quality-marker selection, and extract standardization. Bioactivity data were interpreted in relation to recovery conditions, chemical profiles, and assay contexts rather than as isolated activity endpoints. Finally, application pathways were qualitatively appraised according to extract identity, safety, stability, formulation compatibility, regulatory feasibility, reported scalability, and sustainability-related evidence. To make the evidence hierarchy explicit from the beginning of the review, application-oriented valorization is interpreted as an evidence continuum rather than as a direct transition from extract composition or in vitro activity to application claims. In this continuum, compositional characterization defines what is present in the extract; in vitro activity provides initial screening evidence; mechanism-oriented assays and dose–response analysis strengthen functional interpretation; bioaccessibility and bioavailability studies clarify exposure relevance where appropriate; formulation and stability studies test whether activity-relevant profiles can be retained under use conditions; in vivo or model-system validation provides higher-level biological support where relevant; and sector-specific application readiness integrates identity, safety, stability, formulation, regulatory, scale-up, techno-economic, and sustainability evidence. This hierarchy was used to calibrate claim strength throughout the review and to distinguish screening-level, profile-supported, candidate-validated, and application-oriented evidence.

**Figure 1:**
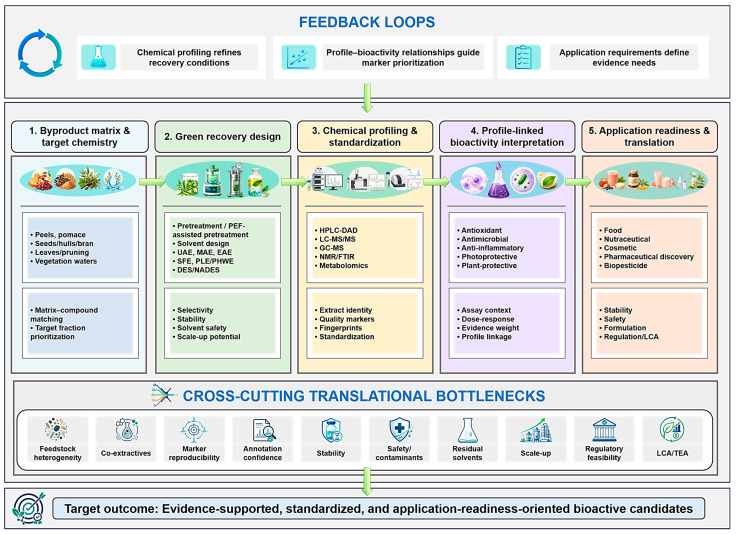
Linkage-oriented framework for circular valorization of agri-food byproduct-derived bioactive compounds.

This framework was used to identify where evidence connections are strong and where gaps remain from feedstock selection to application-oriented valorization. In particular, the synthesis examined whether reported recovery methods generated chemically defined or transparently annotated extracts, whether profiling data supported reproducible marker selection, whether bioactivity claims were linked to compound-level or fingerprint-level evidence, and whether proposed applications were supported by readiness criteria relevant to the intended sector. By emphasizing these connections, this review seeks to move beyond a descriptive summary of byproduct sources and applications and to provide a basis for identifying promising matrix–recovery–profiling–valorization combinations, as well as bottlenecks that limit practical translation. The overall framework is summarized in Figure 1.

The framework links byproduct matrix and target chemistry, green recovery design, chemical profiling and standardization, profile-linked bioactivity interpretation, and application-readiness assessment. Main arrows show the evidence flow from feedstock selection to application-oriented valorization, whereas the upper feedback-loop panel highlights iterative refinement of recovery conditions, marker prioritization, and evidence needs. Cross-cutting bottlenecks include feedstock heterogeneity, co-extractives, marker reproducibility, annotation-confidence limitations, stability loss, safety/contaminant issues, residual solvents, scale-up constraints, regulatory feasibility, and LCA/TEA limitations. The target outcome is evidence-supported, standardized, and application-readiness-oriented bioactive candidates. Accordingly, the framework should be read as a staged evidence continuum from compositional characterization and in vitro screening through mechanism-oriented and exposure-relevant validation, formulation/stability testing, in vivo or model-system support where relevant, and sector-specific application-readiness assessment. Abbreviations: DES, deep eutectic solvents; EAE, enzyme-assisted extraction; FTIR, Fourier-transform infrared spectroscopy; GC-MS, gas chromatography–mass spectrometry; HPLC-DAD, high-performance liquid chromatography with diode array detection; LCA, life cycle assessment; LC-MS/MS, liquid chromatography–tandem mass spectrometry; MAE, microwave-assisted extraction; NADES, natural deep eutectic solvents; NMR, nuclear magnetic resonance; PEF, pulsed electric field; PHWE, pressurized hot-water extraction; PLE, pressurized liquid extraction; SFE, supercritical fluid extraction; TEA, techno-economic analysis; UAE, ultrasound-assisted extraction.

## 3. Agri-Food Byproduct Matrices and Major Classes of Bioactive Compounds

A matrix-oriented perspective is essential for circular valorization because agri-food byproducts are not chemically uniform residue categories [1,2,3]. Their botanical origin, tissue architecture, moisture status, lignocellulosic organization, processing history, and storage conditions can shape the localization, abundance, accessibility, and stability of target bioactive compounds [2,3]. These matrix features influence solvent penetration, mass transfer, release of free or bound metabolites, co-extraction of interfering constituents, chemical stability during recovery, and the analytical requirements needed to define extract identity [2,4]. Accordingly, this review assesses valorization potential through matrix–compound matching rather than by the presence of bioactive compounds alone, using matrix characteristics to guide target-compound prioritization, recovery design, fit-for-purpose chemical profiling, and the intended circular valorization pathway. Consistent with the operational definition introduced above, the presence of a compound class is treated as a recovery opportunity rather than as sufficient evidence of bioactivity. Across matrix classes, bioactive relevance is interpreted according to whether recovered molecules or fractions are chemically characterized and linked to biological, profile-related, or application-specific endpoints.

Agri-food byproducts can be organized according to both residue type and matrix chemistry: solid matrices include fruit and vegetable peels, pomace and pressing residues, seed residues, hulls, shells, bran fractions, leaves, stems, and pruning waste, whereas liquid or semi-liquid streams include vegetation waters, olive mill wastewater, and related aqueous processing residues; across these groups, the recoverable chemical space broadly includes polyphenolic fractions, lipophilic constituents, volatile or essential-oil constituents, and macromolecular or structurally associated fractions such as pectin, dietary fibers, polysaccharides, proteins, and peptides [2,3]. Their distribution is matrix-dependent rather than uniform: peels, pomace, seed coats, and pruning residues are frequently prioritized for phenolic-rich fractions; pigment- or lipid-containing residues are more relevant for carotenoids, tocopherols, fatty acids, and related lipophilic markers; aromatic peels, leaves, and herbaceous residues require attention to volatile terpenoids and essential-oil constituents; and seed residues, oilseed meals, hulls, shells, bran fractions, and other fibrous residues often require strategies for bound phenolics, dietary fiber, protein- or peptide-rich fractions, or polysaccharide-rich fractions [2,3,4]. These matrix–compound relationships provide an initial decision layer for recovery priorities, profiling needs, potential valorization routes, and key limitations, as summarized in Table 1.

### 3.1. Fruit and Vegetable Peels

Fruit and vegetable peels should be treated as chemically heterogeneous external tissues rather than as a single byproduct category. As external tissues, peels are commonly associated with protective metabolites and structural components, including polyphenolic fractions, flavonoids, terpenoids, pigments, cuticular waxes, dietary fibers, and pectic polysaccharides [2,3,19]. Citrus peels exemplify volatile- and pectin-rich matrices, with flavanones, polymethoxylated flavones, limonoids, essential-oil constituents, and pectin frequently targeted [20,21]. Apple, mango, pomegranate, onion, and tomato peels are more commonly investigated for phenolics, flavonoids, pigments, carotenoids, and antioxidant-related markers [2,3,19]. This matrix-specific chemistry means that peel valorization should be guided by the dominant target fraction and by the co-extractives that may affect extract identity, stability, sensory quality, safety, and downstream compatibility, as summarized in Table 1.

Peel-derived extracts can support food preservation, functional-food, nutraceutical, cosmetic, and biopesticide-related pathways [3,8,19,21]. However, their application readiness cannot be inferred from total phenolic content or antioxidant capacity alone, because such bulk indices do not define extract identity, marker reproducibility, safety, stability, or sector-specific performance requirements [2,18,32]. Pigments, waxes, sugars, organic acids, bitter compounds, pesticide residues, and odor-active volatiles may enhance functionality in some contexts while limiting sensory, formulation, safety, or regulatory suitability in others [10,21]. Accordingly, recovery should be fraction-specific: hydroethanolic extraction, ultrasound-assisted extraction, microwave-assisted extraction, or pressurized liquid extraction are appropriate starting points for phenolic-rich fractions; hydrodistillation, solvent-free microwave extraction, or supercritical fluid extraction are more suited to volatile or essential-oil-rich peels; and acid-, enzyme-assisted, or green solvent-based extraction can be prioritized when pectin or cell-wall polysaccharides are the target [19,21,33]. For peel matrices, the key decision is therefore not simply how much material is extracted, but whether the selected process enriches the intended fraction while controlling co-extractives, residues, stability, and application-specific quality attributes, as summarized in Table 1.

### 3.2. Pomace and Pressing Residues

Pomace and pressing residues are composite byproduct matrices generated during juice production, winemaking, oil extraction, puree production, and related industrial operations [22,23,29]. Unlike single-tissue residues, they typically contain variable proportions of skins, pulp, seeds, stems, fibrous material, residual sugars, organic acids, proteins, and lipophilic fractions, depending on crop type and processing sequence [22,23]. Grape pomace illustrates a phenolic- and fiber-rich matrix in which phenolic acids, flavan-3-ols, anthocyanins, proanthocyanidins, stilbenes, dietary fiber, and seed-derived lipid or protein fractions may coexist [22]. Apple, tomato, and other fruit-processing pomaces are commonly prioritized for pectin- and fiber-rich fractions, carotenoids, phenolics, fatty acids, residual oils, or other matrix-specific functional constituents [2,23], whereas olive pomace and olive oil processing residues require additional attention to oil sector phenolics, residual oils, fatty acids, and integrated resource-recovery pathways [29]. Thus, pomace valorization should begin with identifying the dominant anatomical fractions and target chemistry rather than treating pomace as a uniform residue category, as summarized in Table 1.

In contrast to more anatomically defined peel residues, many pomace matrices are analytically challenging because bioactive compounds are spatially and chemically partitioned across skins, seeds, pulp, stems, and fibrous fractions in free, conjugated, polymerized, or matrix-bound forms [4,22]. This partitioning affects solvent penetration, mass transfer, selective release, co-extraction of sugars, acids, proteins, pigments, lipids, or fibers, and the chemical stability of recovered compounds [2,4,22]. Accordingly, pomace valorization should be guided by fraction-specific chemical profiling rather than by extraction yield, total phenolic content, or antioxidant capacity alone [2,18,32]. Chromatographic, mass-spectrometric, and complementary fingerprinting approaches are needed to define whether a recovered material is phenolic-rich, pigment-rich, pectin- or fiber-rich, lipid-rich, or mixed; to compare recovery methods; to monitor marker reproducibility; and to assess whether the resulting extract meets application-specific requirements for identity, stability, sensory or formulation compatibility, and safety [2,4,24].

### 3.3. Seed Residues, Hulls, Shells, and Bran Fractions

Seed residues, hulls, shells, and bran fractions should be considered structurally protected matrices rather than simple extractable residues. They are generated during fruit processing, cereal milling, oilseed processing, nut processing, and legume processing, and their chemical value depends on the dominant anatomical fraction: seed coats, hulls, shells, and bran layers are often enriched in lignocellulosic materials, dietary fiber, tannins, and bound phenolics, whereas kernels, germs, defatted meals, and oilseed residues may provide tocopherols, phytosterols, unsaturated fatty acids, proteins, peptides, polysaccharides, and lipid-associated bioactives [2,3]. In these matrices, target compounds may be physically entrapped or chemically associated with cell-wall polymers, protein networks, or lipid-rich compartments, limiting their accessibility under mild solvent extraction conditions [2,15,25]. Cereal bran illustrates this constraint particularly well because hydroxycinnamic acids, especially ferulic acid, are frequently present in bound forms associated with arabinoxylan- and lignocellulose- rich structures and may require enzymatic, alkaline, fermentation-assisted, or other process- intensified pretreatments for efficient release [15,16,25]. Thus, valorization of seed-, hull-, shell-, and bran-derived materials should begin by distinguishing free, lipid-soluble, protein-associated, and cell-wall-bound fractions before selecting a recovery strategy, as summarized in Table 1.

For these matrices, pretreatment is often not an auxiliary step but a determinant of extract chemistry [2,15,25]. Milling, particle-size reduction, defatting, enzymatic hydrolysis, ultrasound-assisted pretreatment, microwave treatment, fermentation, and solvent engineering can improve matrix disruption, mass transfer, and release of target compounds, but they can also alter the recovered profile by increasing the co-extraction of proteins, lipids, sugars, antinutritional factors, or degradation products [2,15,25]. Process intensity should therefore be controlled to avoid oxidation of unsaturated lipids and tocopherols, excessive hydrolysis of proteins or polysaccharides, loss of thermolabile constituents, or formation of poorly characterized mixtures [2,15]. Phenolic-rich fractions require profiling strategies that distinguish free, released, and bound phenolics; lipid-rich fractions require recovery and quality control focused on tocopherols, phytosterols, fatty acids, and oxidation indicators; and protein—or peptide-rich fractions require attention to degree of hydrolysis, molecular-weight distribution, digestibility, bitterness, potential allergenicity where relevant, sensory acceptability, and safety [2,10,25,26]. For seed residues, hulls, shells, and bran fractions, successful valorization therefore depends less on maximum extraction yield than on whether pretreatment and recovery selectively enrich the intended fraction while preserving chemical integrity, limiting undesirable co-extractives, and supporting application-specific quality control.

### 3.4. Pruning Waste, Leaves, Stems, and Vegetation Waters

Pruning waste, leaves, stems, and vegetation waters represent non-conventional byproduct streams whose valorization requires different logic from that applied to facility-generated solid processing residues. Field-origin materials such as orchard, vineyard, and olive-grove pruning residues are shaped by cultivar, season, agronomic practice, environmental exposure, tissue age, and post-collection handling [27,28,29]. In contrast, liquid or semi-liquid streams such as vegetation waters and olive mill wastewater are shaped by processing conditions, water content, organic load, microbial susceptibility, colloidal complexity, and soluble compound distribution [29,30,31]. Leaves, stems, and pruning residues may accumulate defense-related phenolics, flavonoids, secoiridoids, terpenoids, triterpenes, lignans, alkaloids, and other specialized metabolites, whereas vegetation waters and related liquid residues are more relevant for soluble phenolics, organic acids, soluble or colloidal process-derived constituents, and occasional dispersed lipophilic constituents when supported by profiling [27,29,30]. Thus, these matrices should not be treated as a residual extension of fruit- or pomace-based valorization, but as distinct feedstocks requiring matrix-specific stabilization, recovery, concentration, and chemical profiling strategies.

Olive sector byproducts illustrate this distinction particularly well. Olive leaves and pruning residues are tissue-based matrices in which secoiridoids and related phenolics, especially oleuropein and hydroxytyrosol derivatives, may coexist with triterpenes, flavonoids, lignocellulosic materials, and other structural or lipophilic fractions [27,29]. In contrast, olive mill wastewater and vegetation waters are aqueous effluents enriched in soluble phenolics and organic acids but complicated by high water content, organic load, colloidal complexity, microbial instability, and potential environmental toxicity [29,30,31]. These materials may support food, nutraceutical, cosmetic, pharmaceutical, plant-protective, biopesticide-related, or agricultural valorization routes, depending on matrix type and evidence level; however, their translation requires more than demonstrating phenolic richness or antioxidant activity [2,18,32]. Solid field residues require drying or stabilization, particle-size control, and fraction-specific extraction, whereas liquid streams often require clarification, adsorption, membrane-based separation, resin-based concentration, solvent-assisted recovery, or integrated treatment—biorefinery approaches to couple phenolic recovery with detoxification, concentration, and safe conversion into application-ready fractions [27,30,31]. For pruning residues, leaves, stems, and vegetation waters, the key valorization challenge is therefore to match the physical state and chemical distribution of the matrix with a recovery strategy that preserves target compounds while controlling variability, organic load, contaminants, and downstream processing constraints, as summarized in Table 1.

### 3.5. Matrix–Compound Class Relationships and Implications for Valorization

The matrix–compound class relationship should be interpreted as a practical decision layer for agri-food byproduct valorization rather than as a simple classification scheme. Across the matrices discussed above, peels are often prioritized for phenolic-, flavonoid-, volatile-, pigment-, wax-, and pectin-rich fractions; pomace and pressing residues for polyphenolic, pigment-associated, lipid-associated, pectin- or fiber-rich, and residual seed- or pulp-derived fractions; seed residues, hulls, shells, and bran fractions for bound phenolics, tocopherols, phytosterols, oils, proteins, peptides, polysaccharides, and dietary fiber; pruning waste, leaves, and stems for defense-related phenolics, terpenoids, secoiridoids, lignans, and other specialized metabolites; and vegetation waters or related liquid residues for soluble phenolics, organic acids, aqueous-phase metabolites, and process-derived constituents [2,3]. These associations are best regarded as matrix-specific tendencies or recovery hypotheses rather than fixed rules, because cultivar or genotype, geographic origin, environmental and agronomic conditions, harvest stage and tissue maturity, postharvest handling, industrial processing sequence, storage conditions, pretreatment, and extraction parameters can substantially reshape moisture status, marker abundance, matrix binding, degradation state, extractability, and the final chemical profile [2,4]. For this reason, raw-material variability should be considered an upstream standardization constraint rather than only a source descriptor. Source-to-extract traceability should therefore record crop identity, cultivar or genotype where available, geographical origin, agronomic and environmental context, harvest stage or maturity, storage duration and conditions, industrial processing history, and the pooling or batch logic used before recovery and profiling.

**Figure 2:**
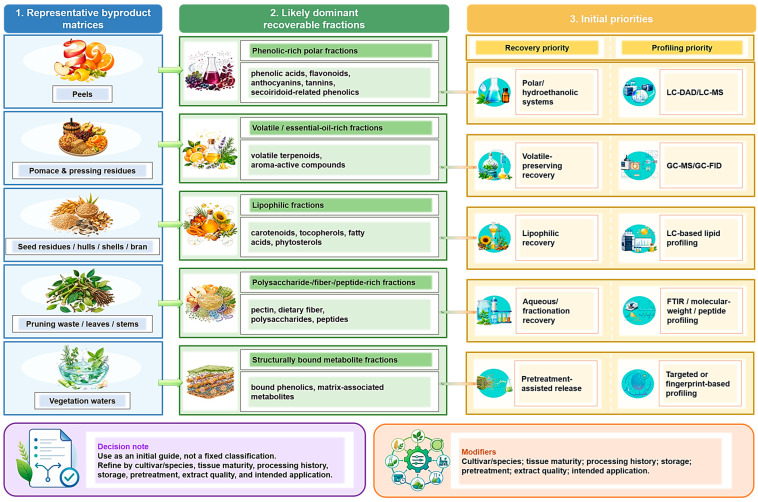
Matrix–fraction decision map for selecting initial recovery and profiling priorities in agri-food byproduct valorization.

In this review, byproduct matrices are treated as decision points that guide target-compound prioritization, recovery design, analytical platform selection, quality-marker definition, safety assessment, and application-route selection. Establishing matrix–compound–recovery relationships can help identify whether a given residue is more suitable for polar phenolic recovery, volatile or essential-oil-oriented valorization, lipophilic compound recovery, polysaccharide- or peptide-rich fractionation, aqueous-phase phenolic recovery, or release of structurally bound metabolites. However, these initial assignments should be refined through fit-for-purpose chemical profiling because extract identity, marker reproducibility, co-extracted constituents, stability, and application-specific requirements ultimately determine valorization potential. The matrix–compound relationships that guide initial recovery and profiling priorities are summarized in Figure 2.

Representative byproduct matrices are linked to likely dominant recoverable fractions and initial recovery and profiling priorities. The horizontal alignment is intended to illustrate common decision paths and should not be interpreted as an exclusive one-to-one assignment between a matrix and a fraction class. Phenolic-rich fractions are commonly approached using polar or hydroethanolic systems and LC-DAD or LC-MS profiling; volatile and essential-oil-rich fractions require volatile-preserving recovery and GC-MS or GC-FID characterization; lipophilic fractions require recovery and profiling strategies suitable for carotenoids, tocopherols, fatty acids, phytosterols, and oxidation markers; polysaccharide-, fiber-, and peptide-rich fractions require aqueous or fractionation-oriented recovery with FTIR, molecular-weight, or peptide-oriented profiling; and structurally bound metabolites may require pretreatment-assisted release before targeted or fingerprint-based profiling. Liquid or semi-liquid residues, such as vegetation waters, require aqueous-effluent-oriented recovery, soluble-marker profiling, residual organic-load monitoring, and safety or reuse assessment. Final recovery and profiling priorities should be refined according to matrix origin, cultivar or species, tissue maturity, processing and storage history, pretreatment, target fraction, extract quality, and intended application.

## 4. Green Recovery Strategies: Selectivity, Efficiency, and Sustainability

Green recovery strategies are central to circular valorization because the recovery step defines not only the amount of material extracted but also the selectivity, chemical integrity, co-extracted matrix components, and downstream usability of the recovered fraction [2,12]. In this review, green recovery is used as a broader concept than green extraction: it includes matrix pretreatment, solvent-system design, process intensification, extraction, concentration or purification where relevant, and post-extraction considerations that influence the selective, safe, and sustainable recovery of bioactive compounds from agri-food byproducts. Accordingly, recovery performance should be assessed through an integrated set of chemical, process, and translational criteria—including target-compound selectivity, compound stability, solvent and process safety, energy and solvent demand, scalability, regulatory acceptability, and application readiness—rather than by extraction yield alone [2,9,34]. For industrial translation, these criteria should also include pilot-scale operability, material and energy balances, solvent recovery and reuse, process throughput, equipment compatibility, waste-stream management, and reproducibility under realistic operating windows. Thus, green recovery technologies are interpreted here not only as extraction tools, but also as process candidates whose scalability depends on downstream integration, solvent and water loops, energy distribution, and techno-economic feasibility.

The matrix–compound relationships discussed in Section 3 provide the starting point for selecting an appropriate recovery strategy. Polar phenolic-rich fractions from peels, pomace, pruning residues, or bran may be approached using hydroethanolic solvent systems, ultrasound-assisted extraction, microwave-assisted extraction, pressurized liquid extraction, or enzyme- or fermentation-assisted release, depending on whether the target compounds are free, conjugated, polymerized, or structurally bound [2,9,15,25]. In contrast, lipophilic carotenoids, tocopherols, fatty acids, phytosterols, and volatile or essential-oil constituents require recovery designs that account for low polarity, oxygen and light sensitivity, volatility, thermal stability, and solvent-removal constraints; supercritical fluid extraction, non-polar or bio-based solvent systems, solvent-free microwave extraction, or volatile-preserving approaches may therefore be more appropriate depending on the target fraction [2,33,35]. Structurally protected matrices such as seed residues, hulls, shells, bran fractions, and fibrous residues may require milling, defatting, enzymatic hydrolysis, alkaline treatment, fermentation-assisted recovery, pulsed electric field treatment, or other process-intensified pretreatments to improve accessibility before extraction [9,15,16,25]. Thus, green recovery should be viewed as a matrix- and compound-class-specific design decision that shapes extract identity, profiling requirements, bioactivity interpretation, and application-readiness outcomes, as summarized in Table 2. A comparative overview of major green recovery strategies, suitable targets, advantages, limitations, and scale-up considerations is provided in Table 2.

### 4.1. Conventional Solvent Extraction as a Reference Baseline

Conventional solvent extraction, including maceration, solid–liquid extraction, reflux extraction, and Soxhlet extraction, remains useful as a reference baseline for evaluating the recovery of bioactive compounds from agri-food byproducts. These methods are operationally simple, broadly accessible, and useful for benchmarking emerging green recovery technologies, particularly when extraction behavior, solvent effects, or matrix–compound accessibility need to be compared under well-established conditions [2,4,36]. However, their practical use is often constrained by prolonged extraction times, high solvent-to-solid ratios, repeated extraction cycles, elevated thermal input, and energy- or solvent-intensive operation, which can reduce their sustainability and limit their suitability for application-oriented valorization [2,3].

The main limitation of conventional solvent extraction is not only its environmental burden but also its limited control over extract chemistry [2,4]. Long residence times, elevated temperatures, oxygen or light exposure, and solvent systems that are poorly matched to the target fraction can promote oxidation, hydrolysis, isomerization, volatilization, or degradation of sensitive constituents such as anthocyanins, carotenoids, tocopherols, volatile terpenoids, and other thermolabile or oxidation-sensitive compounds [2,3,41]. Conventional extraction may also increase the co-extraction of sugars, organic acids, proteins, waxes, pigments, lipids, and other matrix components that influence extract identity, sensory quality, stability, formulation behavior, and downstream compatibility [2,4]. Therefore, conventional extraction should be retained as a useful comparative baseline or as a selected recovery option when justified by target chemistry and solvent safety, but it should not be treated as the default strategy for sustainable valorization. Its results are most informative when solvent composition, extraction time, temperature, solid-to-liquid ratio, pretreatment, yield basis, solvent recovery, and chemical profiling data are reported sufficiently to allow comparison with greener and more selective recovery approaches, as summarized in Table 2.

### 4.2. Ultrasound- and Microwave-Assisted Extraction

Ultrasound-assisted extraction (UAE) and microwave-assisted extraction (MAE) are widely used process-intensified strategies for recovering bioactive compounds from agri-food byproducts, but they should be regarded as distinct recovery designs rather than interchangeable alternatives to conventional extraction [2,9,42]. UAE enhances solvent penetration, matrix disruption, and mass transfer mainly through acoustic cavitation, whereas MAE accelerates compound release through dielectric heating, rapid solvent–matrix interactions, and internal pressure development in microwave-responsive systems [2,42]. Both approaches can reduce extraction time and solvent demand and have been applied to phenolic-, flavonoid-, anthocyanin-, pigment-, pectin-rich, and selected volatile-associated or carotenoid-containing fractions, provided that solvent composition, water content, particle size, energy input, temperature, and extraction time are matched to the target chemistry and matrix structure [2,3,9]. However, process intensification can also reshape extract quality: excessive acoustic power, prolonged sonication, high microwave power, non-uniform heating, or insufficient temperature control may increase oxidation, local thermal stress, isomerization, volatilization, or degradation of thermolabile and oxidation-sensitive constituents [2,42]. Therefore, UAE and MAE should be optimized not only for extraction yield or total phenolic content, but also for target-compound selectivity, preservation of chemical markers, control of co-extracted matrix components, solvent and process safety, scalability, and downstream compatibility, as summarized in Table 2.

### 4.3. Enzyme-Assisted and Fermentation-Assisted Recovery

Enzyme-assisted extraction (EAE) and fermentation-assisted recovery are particularly relevant for structurally protected agri-food byproduct matrices in which target compounds are physically entrapped, cell-wall-associated, protein-bound, glycosylated, polymerized, or linked to lignocellulosic networks [13,15,16]. Rather than functioning only as mild extraction aids, these approaches can modify matrix architecture and, in some cases, transform the chemical forms of recoverable compounds [13,16]. Cellulases, hemicellulases, pectinases, proteases, tannases, β-glucosidases, and esterases can weaken polysaccharide-, pectin-, protein-, glycosidic-, or ester-linked barriers, thereby improving the release of bound phenolics, peptides, polysaccharides, oligosaccharides, and related bioactive fractions under relatively mild conditions [2,9,16]. Fermentation-assisted recovery can further enhance valorization by combining matrix disruption with microbial biotransformation, including the release of bound phenolics, conversion of glycosylated or polymerized metabolites, generation of new bioactive or sensory-active compounds, improvement of protein digestibility, and reduction in selected antinutritional factors [13,15,16].

These advantages, however, depend strongly on biological and process control. Enzyme specificity, dosage, reaction time, pH, temperature, substrate moisture, microbial strain, inoculum conditions, contamination control, and downstream stabilization can determine whether the process selectively enriches the intended fraction or generates poorly characterized mixtures, degradation products, off-flavors, residual enzyme activity, or safety concerns [13,15,16]. Because enzymatic and fermentation-assisted processes can substantially reshape extract composition, they should be coupled with targeted and untargeted chemical profiling to distinguish release from transformation, monitor marker reproducibility, detect undesirable co-products, and interpret bioactivity in relation to the recovered chemical profile [2,6,15]. For these biologically mediated recovery strategies, application readiness therefore depends not only on improved extraction efficiency, but also on controlled biotransformation, compositional traceability, safety, scalability, and compatibility with the intended valorization pathway, as summarized in Table 2.

### 4.4. Supercritical Fluids, Pressurized Liquids, Pulsed Electric Fields, and Green Solvents

Supercritical fluid extraction (SFE), pressurized liquid extraction (PLE), pressurized hot-water or subcritical-water extraction, pulsed electric field (PEF)-assisted pretreatment, and DES/NADES-based green solvent systems expand the recovery toolbox for agri-food byproducts whose target compounds differ widely in polarity, volatility, thermal sensitivity, matrix location, and solvent compatibility. These approaches should not be viewed as interchangeable “green” alternatives, but as matrix- and compound-class-specific strategies that modify mass transfer, solvent selectivity, compound stability, and downstream processing requirements [2,9]. Supercritical CO_2_ is particularly suited to lipophilic and volatile fractions, including carotenoids, tocopherols, fatty acids, phytosterols, terpenoids, and essential-oil constituents, while the use of polar co-solvents can extend its applicability toward moderately polar compounds; however, high-pressure operation, co-solvent selection, equipment cost, solvent recycling, and scale-up feasibility must be considered when SFE is proposed for application-oriented valorization [2,35,43].

PLE and pressurized hot-water or subcritical-water extraction can improve solvent penetration, diffusion, and recovery efficiency by operating at elevated temperature and pressure, but these same conditions may promote hydrolysis, oxidation, isomerization, Maillard-type changes, or thermal degradation when thermolabile phenolics, anthocyanins, carotenoids, tocopherols, or volatile constituents are targeted [2,37,38]. PEF-assisted extraction is more appropriately understood as a non-thermal or mild pretreatment that enhances cell permeabilization and mass transfer before subsequent extraction, particularly in wet, pigment—rich, aromatic, or oil-bearing plant matrices; its effectiveness depends on electric field strength, pulse number, specific energy input, tissue conductivity, moisture content, and integration with a compatible solvent system [2,39]. Deep eutectic solvents (DESs) and natural deep eutectic solvents (NADESs) offer tunable polarity, hydrogen bonding capacity, and solvent–solute interactions for recovering phenolics, flavonoids, anthocyanins, pectins, polysaccharides, and other polar or semi-polar bioactive fractions from food and agro-industrial residues [14,34]. Nevertheless, DES/NADES-based recovery should be evaluated beyond extraction efficiency because viscosity, dilution effects, solvent recovery, residual solvent control, toxicological or ecotoxicological uncertainty, safety assessment, regulatory acceptance, and downstream purification remain decisive constraints for application-ready valorization [14,34]. Thus, these advanced recovery systems are most valuable when selected according to target chemistry, matrix state, process intensity, solvent-removal feasibility, profiling requirements, and the intended application pathway rather than as universally superior alternatives to conventional extraction, as summarized in Table 2.

### 4.5. Recovery Strategy Selection According to Compound Class and Application Readiness

The selection of a green recovery strategy should begin with the intended chemical fraction and application context rather than with the extraction technology itself, as summarized in Table 2. Matrix type, target compound class, molecular form, extraction mechanism, solvent compatibility, process intensity, chemical stability, and downstream requirements jointly determine whether a recovery method can generate an extract of practical value [2,9,11]. In this context, extraction yield alone is an insufficient indicator of valorization success. A high-yield extract may be less valuable if it contains labile compounds, undesirable co-extractives, safety-relevant constituents, residual solvents, excessive pigments, off-flavor precursors, poorly annotated features, or components that compromise sensory quality, formulation behavior, safety, or regulatory suitability [2,4,10,11]. Conversely, a lower-yield fraction may be more suitable for circular valorization when it has a defined chemical identity, reproducible marker profile, demonstrated stability, an acceptable solvent and safety profile, and compatibility with the intended application [2,6,11].

Compound-class matching can provide an initial guide, but it should be treated as a decision framework rather than a fixed hierarchy of extraction methods. Polar phenolic-rich matrices, including peels, pomace, pruning residues, and bran fractions, may be approached using hydroethanolic systems, UAE, MAE, PLE, EAE, or fermentation-assisted recovery depending on whether the target phenolics are free, conjugated, polymerized, or cell-wall-bound [2,9,15,16,25]. Lipophilic or volatile-rich residues require recovery designs that account for low polarity, oxygen and light sensitivity, volatility, thermal stability, and solvent-removal constraints; SFE, non-polar food-grade or bio-based solvent systems, solvent-free microwave extraction, or volatile-preserving approaches may therefore be preferred depending on the target fraction [2,33,35]. Polysaccharide-, fiber-, protein-, or peptide-rich byproducts may require aqueous extraction, enzymatic hydrolysis, fermentation-assisted recovery, or fractionated recovery strategies to balance release efficiency with molecular integrity and sensory or safety considerations [2,15,25,26]. These initial assignments should be verified by fit-for-purpose chemical profiling because recovery conditions reshape marker composition, co-extracted constituents, stability, and bioactivity interpretation [2,6,44]. Overall, green recovery should be viewed as an upstream design gate that links matrix chemistry to extract identity, application readiness, and circular valorization potential rather than as a yield-maximization step, as summarized across Table 2 and further discussed in the application-readiness and translational-bottleneck sections below.

## 5. Chemical Profiling Platforms and Quality Markers

Chemical profiling provides the evidence needed to define heterogeneous agri-food byproduct-derived extracts as chemically interpretable, comparable, and standardizable materials [2,4]. This role is particularly important because green recovery conditions can reshape compound selectivity, marker composition, degradation products, co-extracted matrix components, and downstream usability [2,6,38]. Bulk indicators such as extraction yield, total phenolic content, total flavonoid content, or antioxidant capacity are useful for preliminary screening, but they cannot establish extract identity, annotation confidence, marker reproducibility, contaminant-related risks, or profile–bioactivity relationships [10,18,32,45,46]. Therefore, compound-level quantification, class-specific profiling, untargeted fingerprinting, and marker-based quality control should be integrated into the recovery–valorization workflow rather than added after extraction as a generic descriptive step [2,4,6,44].

The selection of a profiling platform should be guided by target chemistry, matrix complexity, recovery strategy, and intended valorization pathway, as summarized in Table 3. Phenolic- and pigment-rich extracts generally require chromatographic and mass-spectrometric workflows capable of resolving structurally related metabolites, conjugated derivatives, polymeric forms, isomers, and degradation products [2,4,24,44]. Volatile or essential-oil-rich fractions require GC-based profiling supported by retention-index and spectral evidence [47,48,49], whereas lipid-rich fractions require methods suitable for carotenoids, tocopherols, fatty acids, phytosterols, and oxidation-related markers [2,6,41]. Polysaccharide-, fiber-, protein-, or peptide-rich fractions may require complementary spectroscopic, chromatographic, molecular weight, monosaccharide composition, or sequence oriented approaches [2,25,26]. In parallel, NMR, FTIR, NIR, Raman, and other spectroscopic fingerprinting methods, particularly when combined with chemometrics, can support extract comparison, batch classification, and quality-marker selection when compound-level identification is incomplete or not required for the intended application [6,50,51]. Thus, chemical profiling should be designed as a fit-for-purpose analytical strategy that combines targeted markers, fingerprint-level evidence, positive quality markers, and risk-related negative markers to support extract identity, comparison, standardization, and application-readiness assessment, as summarized in Table 3. The main profiling platforms relevant to agri-food byproduct-derived bioactive extracts are compared in Table 3.

### 5.1. Chromatographic Profiling for Phenolics, Flavonoids, and Pigments

HPLC-DAD and UHPLC-DAD remain practical first-line platforms for routine profiling and quantification of UV–Vis-absorbing metabolites in agri-food byproduct-derived extracts [2,4,24]. They are particularly useful for phenolic- and pigment-rich fractions from peels, pomace, seed residues, bran fractions, and pruning residues, where known or expected markers can be monitored across matrices, recovery conditions, and batches [2,4,24]. When authentic standards and validated calibration procedures are available, these platforms provide retention-time matching, UV–Vis spectral information, peak-area-based quantification, peak-pattern comparison, and routine quality control for compounds such as phenolic acids, flavonols, flavan-3-ols, anthocyanins, stilbenes, selected tannin-related markers, secoiridoids, selected carotenoids, and other pigment-related constituents. However, DAD-based evidence should be interpreted primarily as marker-level or class-supporting information in complex extracts, because co-eluting compounds, isomeric structures, polymeric tannins, glycosylated or acylated derivatives, degradation products, and unavailable standards can limit confident structural assignment [2,4,44].

The main value of chromatographic profiling is that it moves extract evaluation beyond global spectrophotometric and bulk indices, including total phenolic content, total flavonoid content, and antioxidant capacity [2,18,32,46]. These indices are useful for rapid screening and method comparison, but they should be interpreted cautiously. The Folin–Ciocalteu assay, commonly used for total phenolic content, reflects overall reducing capacity rather than phenolic specificity and may be influenced by non-phenolic reductants, co-extracted sugars, organic acids, ascorbate, pigments, Maillard-type products, proteins, and other matrix-derived compounds. Total flavonoid assays are also affected by reagent chemistry, calibration standards, wavelength selection, matrix color or turbidity, and the flavonoid subclasses that dominate the extract. Therefore, differences in calibration equivalents, extraction basis, reaction conditions, concentration units, dry-matter correction, and statistical treatment can limit comparability across datasets. For this reason, total phenolic content, total flavonoid content, and related antioxidant indices should be treated as screening-level descriptors rather than as direct proxies for biological activity or application readiness. Phenolic acids, flavonols, flavan-3-ols, anthocyanins, stilbenes, tannin-related markers, secoiridoids, and carotenoid pigments differ in polarity, spectral behavior, stability, sensory contribution, and application suitability, even when they contribute to the same broad “phenolic” or “pigment” category [2,4,41]. Therefore, HPLC-DAD/UHPLC-DAD is well suited for comparing extraction methods, following marker reproducibility, detecting major profile shifts, and monitoring degradation or stability-related changes in known compounds, as summarized in Table 3. Nevertheless, identification based only on retention time and UV—Vis spectra is insufficient when structurally similar metabolites co-elute, when unknown peaks dominate the profile, or when marker compounds must support biological interpretation or application-oriented standardization [44,52]. In such cases, HPLC-DAD/UHPLC-DAD should be complemented with LC-MS/MS, LC-HRMS, authentic standards, or orthogonal fingerprinting approaches to strengthen structural confirmation, annotation confidence, and extract standardization [2,44,52].

### 5.2. Mass Spectrometry-Based Identification and Metabolite Annotation

LC-MS/MS and LC-HRMS can provide more structurally informative evidence than chromatographic retention and UV–Vis spectra alone, making them central to the characterization of chemically complex agri-food byproduct-derived extracts [2,44,52]. Accurate mass, isotope distribution, adduct patterns, precursor/product ion relationships, neutral losses, and fragmentation behavior can support the annotation of phenolics, flavonoid glycosides, selected tannin-related features, secoiridoids, carotenoid derivatives, lipid-associated metabolites, and other specialized compounds that are difficult to resolve or assign confidently using DAD-based methods alone [2,4,44,52]. Targeted LC-MS/MS is particularly useful for marker-based quantification and batch-level standardization when reference compounds, calibration strategies, internal standards, and validation parameters are available [2,24,44]. Untargeted LC-HRMS and metabolomics workflows can reveal chemical differences among byproduct matrices, cultivars, pretreatments, recovery methods, storage conditions, and degradation states, thereby supporting discovery-oriented profiling and candidate-marker prioritization [6,44,52,53].

Annotation confidence should be treated as a central quality criterion in LC-MS-based profiling rather than as a secondary reporting detail [44,52,56]. Confirmed identifications based on authentic standards and matched retention time, accurate mass, and MS/MS spectra should be clearly distinguished from putative annotations based on accurate mass, fragmentation behavior, retention behavior, spectral-library matching, database searching, or in silico evidence [44,52,56]. This distinction is particularly important for byproduct-derived extracts, where isomers, glycosylated or acylated derivatives, polymeric phenolics, in-source fragments, adducts, degradation products, and co-eluting matrix components may lead to over-assignment of tentative features as confirmed compounds [44,52,53]. Overstating annotation confidence can weaken profile–bioactivity interpretation, marker selection, batch comparison, and reproducibility [44,52]. Therefore, LC-MS data used for extract standardization or bioactivity interpretation should report sample preparation, clean-up, ionization mode, mass accuracy, MS/MS acquisition conditions, calibration and quantification strategy, internal standards, quality-control samples, blanks, matrix effects, ion suppression, and method validation where relevant [44,56]. In this context, LC-MS-based profiling is most valuable when it links chemical features to transparent confidence levels, reproducible markers, and application—relevant quality control rather than merely expanding the number of annotated peaks, as summarized in Table 3.

### 5.3. GC-MS Profiling of Volatile and Derivatizable Lipophilic Constituents

GC-MS, often complemented by GC-FID for quantitative or semi-quantitative purposes, is most suitable for volatile, semi-volatile, and derivatizable low-molecular-weight constituents in agri-food byproduct-derived extracts [47,48,49]. It is particularly relevant for essential-oil constituents, aroma-active compounds, monoterpenes, sesquiterpenes, aldehydes, alcohols, esters, derivatized organic acids, fatty acid methyl esters, and selected low-molecular-weight lipophilic metabolites from aromatic peels, leaves, pruning residues, seeds, and other plant-derived byproducts [27,47]. In food, cosmetic, and biopesticide-related valorization, GC-MS-based volatile profiles can provide chemical context for aroma and sensory acceptability, antimicrobial or antifungal potential, plant-protective relevance, formulation behavior, volatility-related stability, and odor-related limitations [8,47]. Thus, volatile profiling should be interpreted not simply as a list of detected compounds, but as a chemical fingerprint that can inform both functional potential and application constraints, as summarized in Table 3.

The reliability of GC-MS profiling depends on how compounds are isolated, separated, annotated, and reported [48,49]. Chromatographic resolution, mass spectral quality, retention—index confirmation, calibration strategy, and comparison with authentic standards all influence whether a volatile or semi-volatile feature can be treated as a reliable marker [48,49]. Sample preparation, headspace or distillation conditions, and derivatization where relevant should also be reported because they can reshape volatile fingerprints and derivatizable low-molecular-weight profiles. Mass spectral library matching is useful for preliminary assignment, but it should not be treated as definitive identification without supporting evidence from experimentally determined retention indices, authentic standards, or complementary analytical information [48,49]. This caution is especially important for essential-oil and aroma-rich byproduct extracts, where isomeric terpenoids, co-eluting compounds, thermally labile constituents, oxidation products, and matrix-derived artifacts can lead to over-assignment or misinterpretation [48,49]. For less volatile or thermally sensitive lipophilic compounds, including carotenoids, tocopherols, phytosterols, and some intact lipids, HPLC-DAD, LC-MS, or dedicated lipid-profiling approaches are generally more appropriate than GC-MS unless suitable derivatization or targeted GC methods are validated [2]. Therefore, GC-MS data should be integrated with retention-index-supported annotation, stability assessment, sensory relevance, formulation behavior, safety considerations, and application-readiness criteria rather than interpreted as standalone compositional evidence, as summarized in Table 3 and further considered in the profile-bioactivity and application-readiness sections below.

### 5.4. Spectroscopic Fingerprinting, Chemometrics, and Quality Markers

Spectroscopic fingerprinting and chemometric analysis provide complementary tools for quality control when exhaustive compound-level identification is unnecessary, impractical, or insufficient for routine comparison of complex byproduct-derived extracts [6,50,51]. NMR, FTIR, NIR, Raman, and related spectroscopic approaches can capture global or class-level chemical signatures associated with dominant metabolite groups, functional groups, matrix—derived components, degradation products, or processing-related changes [6,50,54,55]. When combined with chemometric methods such as principal component analysis, hierarchical clustering, partial least-squares-based models, orthogonal projections, or regression approaches, these fingerprints can support batch comparison, sample classification, origin or processing discrimination, stability monitoring, and prioritization of discriminant features [6,50,51]. However, spectroscopic fingerprints should be interpreted as comparative or quality—control evidence rather than direct structural confirmation unless supported by chromatographic, mass-spectrometric, or reference-standard-based data [50,51,55]. Chemometric models should also be transparently reported and appropriately validated, because poorly controlled preprocessing, overfitting, or insufficient batch representation can convert analytical noise into apparent markers [6,44,51].

Quality markers should be selected according to the intended valorization pathway and the attribute that must be controlled, rather than analytical convenience alone, as reflected in Table 3 and further discussed in the later application-readiness and translational-bottleneck sections. A marker may be a specific bioactive compound, a representative compound class, a diagnostic chromatographic or spectroscopic fingerprint, a compound ratio, a degradation product, or a risk-related indicator that reflects extract identity, functional relevance, stability, reproducibility, or safety-relevant attributes [2,6,45,51]. Marker selection should therefore be matrix- and application-specific, as summarized in Table 3: phenolic-rich extracts may require markers for phenolic acids, flavonoids, anthocyanins, stilbenes, secoiridoids, or their degradation products; volatile fractions may require retention-index-supported terpenoid profiles or odor-active fingerprints; lipid-rich fractions may require carotenoids, tocopherols, fatty acids, phytosterols, or oxidation indicators; and peptide-, polysaccharide-, or fiber-rich fractions may require molecular-weight distribution, monosaccharide composition, peptide profiles, amino acid composition, or degree-of-hydrolysis indicators [2,6]. Because crude byproduct-derived extracts may contain desirable bioactives together with safety-relevant constituents, quality control should include both positive markers associated with desired composition or activity and negative or risk-related markers associated with contamination, degradation, instability, or process-related hazards [2,10,45]. These negative markers should cover pesticide residues, mycotoxins, heavy metals, microbial contaminants, allergens, antinutritional factors, residual solvents, natural toxicants, process-derived contaminants, and degradation products formed during storage, recovery, concentration, or formulation. Therefore, fit-for-purpose marker panels should be designed not only to define extract identity and activity-relevant composition, but also to detect safety-relevant hazards according to feedstock origin, processing history, recovery method, concentration factor, intended route of use, and exposure context. A fit-for-purpose marker panel therefore links extract identity, reproducible quality, functional relevance, stability, and safety, rather than relying on a single convenient compound or a single total-index measurement, as summarized in Table 3 and further discussed in the later application-readiness and translational-bottleneck sections.

To support standardization across variable raw materials, marker panels should also be interpreted together with source metadata and batch-level fingerprints. At minimum, studies should report crop identity, cultivar or genotype where available, geographical origin, environmental or agronomic conditions, harvest stage or maturity, storage conditions, and industrial processing history, because these factors can shift marker ranges, degradation indicators, co-extractives, moisture or dry-matter basis, and batch-to-batch reproducibility. Such information allows chemical profiling to move from one-time composition reporting toward source-normalized quality specifications and acceptable marker ranges.

## 6. Linking Extraction Variables to Chemical Profiles and Bioactivity Interpretation

The preceding sections establish that agri-food byproduct valorization depends on matrix-specific recovery strategies and fit-for-purpose chemical profiling. The critical next step is to interpret extraction variables, chemical profiles, and bioactivity data as connected evidence rather than as independent endpoints, as summarized in Table 4. In much of the agri-food byproduct literature, extraction performance is still evaluated through yield, total phenolic content, total flavonoid content, or single antioxidant responses, which can obscure whether a given recovery condition actually enriches relevant markers, preserves labile compounds, limits undesirable co-extractives, or generates a reproducible and application-suitable extract [2,18,32,46]. In practice, solvent system, water content, pH, temperature, extraction time, particle size, solid-to-liquid ratio, pretreatment, oxygen and light exposure, and assisted extraction technology shape compound release, transformation, degradation, and co-extraction; the resulting chemical profile then provides the context for interpreting biological responses [2,6,38]. Therefore, extraction conditions should be treated as determinants of chemical identity and functional evidence rather than as neutral technical parameters.

A linkage-oriented interpretation is particularly important for agri-food byproduct extracts because these materials are chemically heterogeneous and may contain structurally related metabolites, bound or polymerized compounds, degradation products, and matrix-derived co-extractives [2,4,44]. Two extracts with similar yield, total phenolic content, or apparent antioxidant capacity may differ markedly in phenolic subclass distribution, marker reproducibility, lipophilic or volatile constituents, degradation products, residual matrix components, and assay-interfering substances [2,6,18,32]. Bioactivity data should therefore be interpreted together with compound-level or fingerprint-level information, assay context, and extract-quality attributes rather than treated as isolated assay results [2,6]. This approach enables more cautious evidence weighting: profile-supported activity can guide marker selection, fraction prioritization, and application-route decisions, whereas activity reported without adequate chemical characterization should be treated as preliminary screening evidence rather than proof of valorization potential [44,57], as summarized in Table 4.

### 6.1. Extraction Variables as Determinants of Chemical Profiles

Extraction variables should be treated as determinants of extract chemistry rather than as operational settings used only to maximize yield, as summarized in Table 4. Solvent polarity, water content, hydrogen bonding capacity, viscosity, temperature, extraction time, pH, oxygen and light exposure, particle size, solid-to-liquid ratio, and pretreatment collectively influence compound release, solubilization, diffusion, transformation, degradation, and co-extraction [2,34,38]. Hydroethanolic systems are commonly suited to phenolic acids, flavonoids, anthocyanins, and other moderately polar constituents because they balance polarity, hydrogen bonding interactions, and matrix penetration; however, their selectivity depends strongly on water content, pH, extraction time, and temperature [2,4,38]. In contrast, supercritical CO_2_ and co-solvent-assisted systems are more appropriate for lipophilic or volatile fractions, including carotenoids, tocopherols, fatty acids, phytosterols, and terpenoids, when solvent density, pressure, temperature, and co-solvent composition are matched to the target fraction [2,35,43]. Water-rich or pressurized hot-water systems may favor polar phenolics, polysaccharides, peptides, protein-derived fractions, and other hydrophilic constituents, but they may also increase the co-extraction of sugars, organic acids, proteins, salts, and other matrix-derived components that affect extract identity, stability, and downstream compatibility [2,37,38].

Temperature, pretreatment, and process intensity can improve mass transfer, disrupt cell-wall structures, and release bound or polymerized compounds, but the same variables can also reshape the recovered profile if they are not controlled. Excessive heat, prolonged extraction, oxygen or light exposure, extreme pH, aggressive mechanical disruption, or highly reactive solvent systems may promote oxidation, hydrolysis, isomerization, polymerization, volatilization, or degradation of sensitive constituents such as anthocyanins, carotenoids, tocopherols, volatile terpenoids, and selected phenolics [2,38,41,58]. Therefore, extraction variables should be optimized through a profile-guided approach that considers target-compound enrichment, preservation of labile markers, reduction of undesirable co-extractives, solvent and process safety, and compatibility with subsequent profiling, bioactivity testing, and application requirements. In this sense, the most informative extraction condition is not necessarily the one that gives the highest yield, but the one that generates a chemically defined, reproducible, and application-suitable extract, as summarized in Table 2 and Table 4.

### 6.2. Chemical Profiles as Interpreters of Bioactivity

Chemical profiles provide the interpretive bridge between extraction-induced compositional changes and reported bioactivities, as summarized in Table 4. Without compound-level or fingerprint-level characterization, bioactivity data may indicate that an extract is active, but they cannot clarify whether the response is associated with enriched target markers, specific compound subclasses, degradation products, mixture-level effects, or assay-interfering co-extractives [2,4,6]. Bulk indicators such as total phenolic content, total flavonoid content, and antioxidant capacity are useful for preliminary screening and method comparison, but they are method-, calibration-, and matrix-dependent and cannot sufficiently define extract identity, marker distribution, reproducibility, or functional relevance [18,32,46,59]. High total phenolic or flavonoid values may reflect global reducing capacity, reagent-responsive compound classes, or co-extracted matrix components rather than a defined active marker set. Therefore, these indices should be interpreted together with chromatographic, mass-spectrometric, or fingerprint-level evidence rather than used as standalone proxies for valorization potential or biological activity.

Profile-based interpretation is particularly important because extracts with similar total phenolic content or comparable antioxidant responses may differ markedly in phenolic subclass distribution, glycosylation or acylation patterns, polymerized or bound forms, degradation products, lipophilic or volatile co-constituents, and residual matrix components [2,6,18,32]. Such differences can alter antioxidant, antimicrobial, anti-inflammatory, cytoprotective, or plant-protective behavior, as well as stability, sensory properties, formulation compatibility, and application suitability [2,8,24,60]. Chemical profiling should therefore be used to weight bioactivity claims, identify reproducible markers or discriminant fingerprints, prioritize active fractions, and determine whether the observed activity is relevant to the intended application [6,57]. In this context, an extract should not be considered application-ready merely because it shows high total phenolic content or strong in vitro antioxidant activity; it becomes more convincing when the reported activity is supported by a defined chemical profile, reproducible marker evidence, appropriate assay context, and application-specific quality criteria, as summarized in Table 4 and Table 5.

Accordingly, composition–activity relationships in byproduct-derived extracts should not be interpreted as simple one-marker/one-effect relationships. Reported biological responses may reflect additive, synergistic, or antagonistic interactions among phenolics, organic acids, peptides, lipids, volatile constituents, polysaccharides, pigments, degradation products, and co-extracted matrix components. Matrix effects may further influence solubility, partitioning, bioaccessibility, membrane interaction, assay readouts, and apparent potency. Low-abundance constituents can also contribute disproportionately when they are highly active, affect the stability of major markers, or modulate assay responses. Therefore, marker compounds should be used as anchors for interpretation and standardization rather than as exclusive causal explanations of bioactivity unless supported by fractionation, authentic standards, spike-in or depletion experiments, orthogonal assays, or targeted validation.

### 6.3. Assay-Dependent Limitations and Reporting Challenges

Bioactivity interpretation should account for assay dependence, matrix effects, and application context rather than treating assay responses as universal indicators of functional value, as summarized in Table 4. Antioxidant assays such as DPPH, ABTS, FRAP, ORAC, and lipid oxidation models measure different endpoints, including radical scavenging, reducing capacity, peroxyl radical quenching, reaction kinetics, or protection against oxidation in model systems; therefore, their results should not be interpreted as interchangeable evidence of application-relevant antioxidant functionality [32,61]. Antimicrobial assays are similarly sensitive to methodological context, because solubility or dispersion, diffusion behavior, volatility, turbidity, pH, residual solvents or emulsifiers, inoculum size, microbial strain, growth medium, incubation conditions, and endpoint measurement can strongly influence inhibition zones, MIC values, or growth-based readouts [62,63]. In cell-based assays, crude byproduct-derived extracts may confound viability, cytotoxicity, anti-inflammatory, or protective-effect measurements through precipitation, intrinsic color or absorbance, fluorescence quenching, redox activity, interaction with culture media, or direct interference with tetrazolium-based and other viability or metabolic readouts [64,65].

Bioactivity studies should therefore report the extraction conditions, extract concentration basis, chemical profile or fingerprint, assay format, assay medium or matrix, positive and negative controls, solvent or vehicle controls, blank correction, concentration range, dose–response behavior, normalization basis, replicate structure, and statistical treatment [32,62,63,65]. When possible, complementary assays with orthogonal endpoints should be used to distinguish general redox activity, antimicrobial inhibition, cytotoxicity, metabolic modulation, and application-relevant functionality [32,61,65]. Biological endpoints are most informative when they are linked to compound-level markers, fingerprint-level evidence, extract-quality attributes, and the intended application context rather than reported as isolated assay outcomes [2,6,65]. For this reason, strong in vitro responses should be treated as screening evidence unless they are supported by adequate chemical characterization, appropriate controls, dose-dependent behavior, assay relevance, and application-specific validation, as summarized in Table 4 and Table 6.

### 6.4. Integrating Targeted and Untargeted Profiling with Bioactivity Data

Targeted and untargeted profiling should be used as complementary evidence layers rather than as competing analytical options [66]. Targeted profiling is most appropriate when marker compounds or marker classes are known, expected from matrix chemistry, or required for standardization. Examples include flavanones in citrus peels, stilbenes and flavan-3-ols in grape pomace, hydroxytyrosol and secoiridoid derivatives in olive byproducts, anthocyanins in berry residues, carotenoids in tomato or other pigment-rich byproducts, and volatile terpenoids in aromatic or herbaceous residues [2,24,29,67,68]. In such cases, targeted methods can support marker quantification, batch comparison, recovery optimization, and application—oriented quality control, as summarized in Table 4. By contrast, untargeted LC-HRMS, GC-MS, NMR, FTIR/NIR/Raman, and metabolomics-based workflows are more useful when active constituents are unknown, when extract composition is strongly shaped by matrix or process variability, or when discriminatory fingerprints are needed to compare byproduct sources, pretreatments, recovery methods, storage conditions, or bioactivity patterns [6,50,53,54,55,66]. Thus, targeted profiling provides confidence around predefined markers, whereas untargeted profiling expands the chemical search space and can reveal unexpected markers, co-extracted constituents, degradation products, or discriminant features that targeted assays may miss, as summarized in Table 4. However, correlations between individual markers and reported bioactivities should be interpreted as hypotheses rather than proof of causality. Untargeted or semi-targeted profiling can help identify low-abundance or unexpected constituents, co-varying metabolite clusters, degradation products, and matrix-associated features that may contribute to, suppress, or modify the observed biological response. Therefore, profile—bioactivity interpretation is strengthened when targeted marker data are combined with broader fingerprints, fractionation or depletion logic, orthogonal bioassays, and application-relevant controls.

Integrating chemical profiles with bioactivity data is valuable primarily as a hypothesis—generating and evidence-prioritization strategy, not as direct proof of causality. When LC-MS features, volatile fingerprints, NMR or FTIR/NIR/Raman spectral variables, or specific compound subclasses consistently covary with antioxidant, antimicrobial, cell-based, plant-protective, or other bioactivity endpoints, they can be prioritized as candidate markers or active fractions for further testing [24,57,69,70]. However, profile–bioactivity correlation should not be interpreted as mechanistic proof, because apparent associations may arise from co-elution, covariance among chemically related metabolites, matrix effects, assay interference, concentration differences, batch structure, or chemometric overfitting [6,55,57]. Candidate markers identified through chemometric, metabolomics, or multivariate feature-prioritization workflows should therefore be validated using authentic standards, targeted quantification, fractionation, purified compounds, spiking or depletion experiments, orthogonal bioassays, and dose–response testing when mechanistic or application-level claims are intended [44,57,70]. In this review, profile–bioactivity integration is therefore treated as a structured interpretation framework that links extraction variables, chemical profile changes, assay outcomes, and validation requirements rather than as a direct route from statistical correlation to confirmed bioactive compounds. This framework is summarized in Table 4.

**Table 4:** Linkage framework for interpreting extraction variables, chemical profiles, and bioactivity outcomes in agri-food byproduct valorization.

Evidence Layer and Decision Question	Chemical-Profile Evidence to Examine	Bioactivity Interpretation and Risk Control	Minimum Validation and Reporting Requirements	Representative References
Extraction-variable to profile layer Which variables reshape the recovered profile?	Targeted markers; class-specific profiles; fingerprints; co-extractives; degradation products; marker preservation; yield, dry-weight, and concentration basis.	Compare activity only relative to extract identity, marker content, dose/concentration basis, and profile quality. Avoid yield-only or TPC-only claims.	Report solvent system, pH, time, temperature, S:L ratio, particle size, pretreatment, oxygen/light exposure, assisted technology, feedstock basis, yield basis, concentration basis, and chemical-profile data.	[2,6,38]
Bulk-index and preliminary activity screening Are yield/TPC/TFC, IC50, EC50, inhibition %, zones, or MIC only screening evidence?	Total-index or assay-response data, supported where possible by chromatographic, MS, spectroscopic, or fingerprint-level evidence.	Bulk indices can rank early extracts but cannot define identity, marker distribution, reproducibility, mechanism, or application relevance. Method and matrix effects can distort comparisons.	Specify calibration standards, assay conditions, concentration basis, blanks, solvent/vehicle controls, normalization, replicate structure, statistics, and complementary profiling evidence.	[18,32,46]
Targeted profiling of expected markers Are expected markers enriched, preserved, and reproducible?	Reference-standard-supported quantification of predefined compounds/classes, e.g., flavanones, stilbenes, anthocyanins, hydroxytyrosol derivatives, carotenoids, terpenoids, or degradation markers.	Supports optimization, batch comparison, marker specifications, and QC; narrow panels may miss unexpected actives, co-extractives, or mixture effects.	Use calibrated methods, authentic standards where available, internal standards, defined marker ranges, positive and negative quality markers, and a clear quantification basis.	[2,24,29]
Untargeted fingerprinting and discovery profiling Which unexpected features or fingerprints distinguish matrices, processes, storage states, or activity groups?	LC-HRMS, GC-MS, NMR, FTIR/NIR/Raman fingerprints; feature tables; spectral variables; discriminant features; chemometric patterns; annotation-confidence levels.	Expands chemical search space and generates candidate-marker hypotheses; tentative annotations, batch effects, and overfitted models can weaken interpretation.	Include QC samples, blanks, preprocessing details, annotation-confidence reporting, replication and batch structure, model validation, transparent statistics, and validation planning.	[6,53,66]
Assay-context and matrix-effect layer Could assay conditions or matrix interference distort apparent activity?	Solvent/vehicle residues; turbidity; intrinsic color, absorbance, or fluorescence; redox-active co-extractives; pH; precipitation; volatile loss; medium or reagent interference.	Different antioxidant, antimicrobial, cell-based, lipid-oxidation, and plant-protective assays measure different endpoints. Matrix effects can mimic, mask, or exaggerate responses.	Use positive/negative controls, solvent/vehicle controls, blanks, blank correction, concentration ranges, dose–response behavior, assay-medium details, and orthogonal endpoint assays.	[62,63,64]
Profile-bioactivity association/biochemometric layer Which features, fingerprints, or fractions covary with activity?	Marker abundance; LC-MS or GC-MS features; volatile fingerprints; NMR/FTIR variables; multivariate loadings; active fractions; replicate and batch structure.	Associations prioritize hypotheses but are not causality. Co-elution, covariance, matrix effects, assay interference, concentration effects, batch structure, or overfitting can create false markers.	Validate across batches, matrices, or independent datasets where possible. Use cross-validation, permutation/model checks, transparent statistics, and cautious candidate-marker language.	[57,69,70]
Candidate marker or active fraction validation Does the candidate marker, class, or fraction contribute to activity?	Confirmed identity or transparent annotation level; targeted quantification; purified compounds; active fractions; spiking/depletion evidence; degradation controls.	Validation strengthens functional interpretation, but single-compound testing may not capture synergy, antagonism, or matrix-dependent effects.	Use authentic standards, targeted quantification, fractionation, purified compounds, spiking/depletion experiments, orthogonal bioassays, dose–response testing, and mechanism-oriented experiments where needed.	[44,57,70]
Application-route evidence weighting Is the observed activity relevant to the intended sector and use scenario?	Positive and negative quality markers; marker reproducibility; stability/degradation profile; residual solvent or contaminant indicators; formulation or food-matrix compatibility; sector-specific fingerprints.	Weigh activity against extract identity, stability, safety, formulation behavior, exposure/use scenario, and sector-specific performance. Do not transfer activity automatically across sectors.	Define the intended use and evidence level. Report stability, safety, formulation compatibility, application-relevant assay performance, and whether evidence is screening-level, profile-supported, candidate-validated, or application-oriented. Avoid using application-ready unless sector-specific evidence is sufficient.	[2,8,11]

Note: Profile–bioactivity integration is presented as a hypothesis-generating and evidence-prioritization framework rather than as direct proof of causality. Evidence layers indicate decision-support checkpoints rather than fixed requirements; they should be refined according to matrix type, recovery conditions, assay context, annotation confidence, validation goal, and intended application. The rightmost column lists representative references only. Abbreviations: EC50, half-maximal effective concentration; FTIR, Fourier-transform infrared spectroscopy; GC-MS, gas chromatography–mass spectrometry; IC50, half-maximal inhibitory concentration; LC-HRMS, liquid chromatography–high-resolution mass spectrometry; LC-MS, liquid chromatography–mass spectrometry; MIC, minimum inhibitory concentration; MS, mass spectrometry; NIR, near-infrared spectroscopy; NMR, nuclear magnetic resonance; QC, quality control; S:L, solid-to-liquid ratio; TFC, total flavonoid content; TPC, total phenolic content.

### 6.5. Toward Profile-Guided Circular Valorization

Profile-guided circular valorization uses chemical profiles, quality markers, and bioactivity evidence to define the feasible application route of a byproduct-derived extract rather than assuming that activity observed in a generic assay can be transferred directly across sectors, as summarized in Table 4. In this approach, the extract profile functions as an application-decision layer: marker composition, fingerprint reproducibility, co-extracted constituents, degradation products, residual solvent status, stability, and safety-related indicators collectively determine whether a recovered fraction is suitable for food, nutraceutical, cosmetic, pharmaceutical, biopesticide-related, or agricultural use [2,6,10,11,45]. Sector-specific requirements differ substantially. To avoid treating all bioactivity evidence as equivalent, application prioritization is interpreted across practical evidence levels. Screening-level evidence includes compositional characterization and preliminary in vitro activity. Profile-supported evidence requires defined extract identity, marker or fingerprint reproducibility, assay context, and dose–response behavior where possible. Candidate-validated evidence requires stronger biological support, such as mechanism-oriented or orthogonal assays, exposure-relevant information including bioaccessibility or bioavailability where relevant, formulation compatibility, and stability testing. Application-oriented evidence further requires sector-specific safety, regulatory, performance, scale-up, techno-economic, and sustainability support before stronger readiness claims are made. Food applications emphasize food-grade recovery, sensory acceptability, color or oxidative stability, food-matrix interactions, and shelf-life performance [10,71,72]. Nutraceutical and supplement pathways require marker-based standardization, defined chemical identity, documented stability, plausible intake or dose basis, and, where relevant, bioaccessibility or bioavailability evidence [73,74,75]. Cosmetic applications require skin-relevant functionality, photostability, color and odor compatibility, irritation or sensitization-relevant evidence, and formulation behavior [7,11,76,77]. Biopesticide-oriented and agricultural applications require target specificity, phytotoxicity assessment, non-target effects, environmental persistence or degradation behavior, formulation durability, and greenhouse or field relevance [8,78,79].

**Figure 3:**
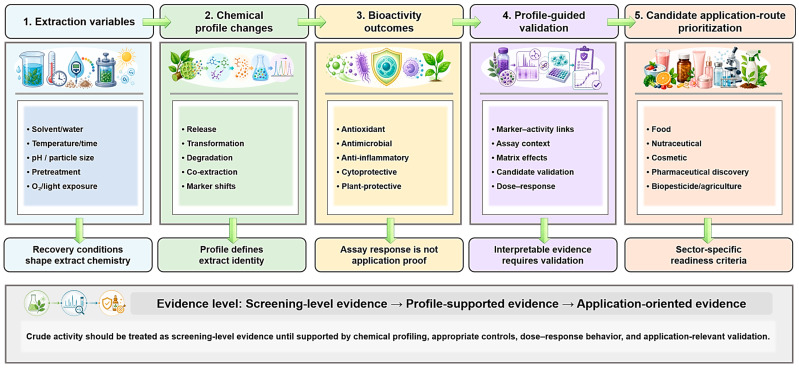
Profile-guided interpretation framework linking extraction variables, chemical profile changes, bioactivity outcomes, and application-route prioritization.

Accordingly, extraction variables should be interpreted as upstream determinants of chemical profiles, while chemical profiles should serve as the basis for weighting bioactivity evidence and selecting application routes, as summarized in Table 4 and Figure 3. A phenolic-rich extract, for example, may be promising for food preservation, nutraceutical standardization, cosmetic antioxidant formulations, or plant-protective applications, but the appropriate pathway depends on whether its marker profile, solvent system, stability, sensory or formulation behavior, safety profile, and validation evidence match the intended use. Similarly, pigment-rich, volatile-rich, lipid-rich, polysaccharide-rich, or peptide-rich fractions require different evidence thresholds before they can be considered application-ready, as reflected in Table 5 and Table 6. Profile-guided validation can therefore reduce overinterpretation of crude bioactivity assays by linking observed activity to extract identity, reproducible markers, assay context, and sector-specific readiness criteria. The extraction-variable-to-bioactivity interpretation framework for profile-guided circular valorization is summarized in Figure 3.

The framework illustrates how extraction variables, including solvent composition and water content, temperature and extraction time, pH, particle size, pretreatment, oxygen or light exposure, and assisted extraction technology, shape byproduct-derived extract profiles through compound release, transformation, degradation, co-extraction, marker preservation, and fingerprint shifts. These chemical profile changes provide the context for interpreting selected bioactivity outcomes, including antioxidant, antimicrobial, anti-inflammatory, cytoprotective, and plant-protective responses. Profile-guided validation evaluates marker–activity links, assay context, matrix effects, candidate validation, and dose–response behavior before prioritizing sector-specific application routes, including food, nutraceutical, cosmetic, pharmaceutical discovery, and biopesticide or agricultural use. The evidence-level bar emphasizes that crude bioactivity should be treated as screening-level evidence until supported by chemical profiling, appropriate controls, dose–response behavior, and application-relevant validation.

## 7. Circular Valorization Pathways and Application Readiness

Circular valorization should be evaluated not only by the recovery of chemically active fractions, but also by whether those fractions can meet application-specific evidence requirements, as summarized in Table 5. A byproduct-derived extract becomes translationally meaningful when its chemical identity, marker reproducibility, stability, safety profile, solvent and formulation compatibility, scalability, regulatory feasibility, and economic and sustainability-related evidence are aligned with a defined use context [2,11,80]. Accordingly, application-route selection should begin at the recovery and profiling stages: the intended sector determines which compounds or fingerprints should be enriched, which co-extractives or contaminants must be controlled, which stability constraints must be addressed, and which performance evidence is required for practical use [4,6,11].

This application-readiness perspective is particularly important because byproduct-derived extracts are chemically complex and compositionally variable. Phenolic-rich extracts, essential-oil-rich fractions, carotenoid-containing extracts, polysaccharide-rich fractions, and peptide-rich hydrolysates may each require different recovery designs, profiling platforms, stabilization approaches, safety evaluations, and formulation strategies before they can be considered suitable for food, nutraceutical, cosmetic, pharmaceutical, or biopesticide-related applications [2,81,82], as summarized across Table 2, Table 3 and Table 5. Thus, application readiness should be treated as an integrated outcome of matrix selection, green recovery, chemical profiling, profile-linked bioactivity interpretation, safety assessment, and intended-sector constraints, rather than as a conclusion inferred from compound presence, extraction yield, or preliminary in vitro activity alone, as summarized in Table 4 and Table 5.

### 7.1. Food and Functional Food Applications

Food and functional food applications represent one of the most direct circular valorization pathways for agri-food byproduct-derived extracts and fractions, but food use should be defined by performance in a specific food matrix rather than by the general presence of bioactive compounds, as summarized in Table 5. Fruit- and vegetable-derived byproducts, including peels, seeds, pomace, and leaves, have also been reviewed as sources of polyphenols, carotenoids, dietary fibers, glucosinolates, phytosterols, and essential-oil constituents with potential food and nutraceutical uses, although standardization, safety assessment, and regulatory approval remain important constraints [83]. Materials recovered from peels, pomace, seeds, bran fractions, and other processing residues may contribute to oxidative stability, color protection, microbial control, flavor or aroma modulation, dietary-fiber enrichment, texture modification, or functional fortification, depending on their dominant chemical fraction and co-extracted components [72,82,84]. In dairy matrices, including cheese and related products, agri-food byproducts have also been examined as functional ingredients, although matrix-specific effects on sensory and technological quality require careful evaluation [85]. Phenolic-rich extracts may be relevant when their marker profile and redox behavior translate into delayed lipid oxidation or microbial inhibition in the target food system, whereas pigment-rich fractions require evaluation of color stability under relevant pH, light, thermal, and storage conditions [41,86,87]. Fiber- or polysaccharide-rich fractions may support water-binding, viscosity, texture, or, in selected matrices, prebiotic-oriented functionality, but these effects depend on molecular structure, purity, dose, and food-matrix interactions [88,89]. Thus, food-oriented valorization requires in-matrix performance evidence rather than extrapolation from crude antioxidant, antimicrobial, or total phenolic assays alone.

Food-grade recovery and formulation compatibility are decisive for translating a chemically active extract into a usable food ingredient. Solvent systems, extraction aids, carriers, stabilizers, encapsulation or drying processes, and residual-solvent profiles should be compatible with food use and with the intended product matrix; water, ethanol, and hydroethanolic mixtures are generally more defensible for food-directed recovery than non-food-grade or analytically convenient organic solvents that are difficult to justify in the final food matrix [10,11,72]. Even when an extract shows strong chemical or biological activity, food incorporation may be limited by non-compliant residual solvents, pesticide residues, microbial or chemical contaminants, excessive pigments, bitter or astringent compounds, odor-active volatiles, turbidity, unstable color, or interactions with proteins, lipids, polysaccharides, minerals, and other food-matrix components [10,71,90]. Candidate extracts should therefore be evaluated under realistic processing, formulation, and storage conditions using product-relevant shelf-life studies, lipid-oxidation or color-stability models, microbial challenge tests when appropriate, sensory evaluation, and assessment of food-matrix interactions or bioaccessibility [71,72,90,91]. For food and functional food applications, the key question is not whether a byproduct-derived extract is active in vitro, but whether its chemical identity, safety profile, sensory and technological behavior, and stability support a defined function in a specific food system, as summarized in Table 5.

### 7.2. Nutraceutical, Supplement, and Pharmaceutical Applications

Nutraceutical, supplement, and pharmaceutical applications should be treated as high-value but evidence-intensive valorization routes rather than direct extensions of preliminary food-application screening, as summarized in Table 5. Agri-food byproduct-derived extracts may contain health-relevant polyphenolic, carotenoid-, tocopherol-, peptide-, polysaccharide-, fatty acid-, terpenoid-, and other specialized metabolite-rich fractions [81,92,93]. However, chemical richness or promising in vitro activity alone does not justify nutraceutical or pharmaceutical translation unless extract identity, exposure relevance, safety, and claim-relevant evidence are established. For these pathways, candidate extracts require defined chemical identity, marker-based standardization, batch-to-batch reproducibility, documented stability, an acceptable safety profile, and biological evidence that is relevant to the intended claim or development stage [73,74,94]. Accordingly, byproduct-derived materials should be framed as standardized extracts, bioactivity-guided fractions, or early-stage discovery candidates, with their target markers, negative quality indicators, residual-solvent status, contaminant profile, and intended biological endpoint clearly defined before stronger application claims are made, as summarized in Table 5.

For nutraceutical and supplement use, reproducible marker content and exposure-relevant performance are particularly important. Extracts should have marker-defined specifications, validated or fit-for-purpose analytical methods, documented stability under storage and formulation conditions, and, when relevant, bioaccessibility or bioavailability data [73,74,75]. Phenolic-rich extracts may undergo digestive transformation and gut microbiota-mediated metabolism, meaning that the compounds detected in the original extract may differ from the species reaching intestinal compartments or contributing to downstream biological effects [75,95]. Carotenoids and tocopherols require appropriate lipid, emulsion, or micellar environments for intestinal uptake, whereas peptide-rich hydrolysates may be further hydrolyzed or structurally modified by gastrointestinal enzymes, affecting both activity and reproducibility [96,97,98]. Pharmaceutical applications require a still higher evidence tier, including mechanism-oriented validation, dose–response assessment, target or pathway relevance, cytotoxicity and broader safety evaluation, pharmacological and toxicological assessment, and in vivo or clinical evidence where relevant [65,94,99]. Thus, these pathways are most realistic when positioned as standardized extract development, bioactivity-guided fractionation, or early-stage natural-product discovery rather than as direct pharmaceutical translation from crude byproduct extracts [92,94,99].

### 7.3. Cosmetic and Dermatological Applications

Cosmetic and dermocosmetic applications are attractive but evidence-sensitive valorization routes because byproduct-derived extracts must function within topical formulations and at the skin interface, as summarized in Table 5. Plant-derived residues can provide polyphenolic, flavonoid-, carotenoid-, tocopherol-, terpenoid-, essential-oil-, polysaccharide-, lipid-, and peptide-rich fractions with potential relevance to antioxidant protection, oxidative-stress modulation, photoprotective support, antimicrobial preservation support, pigmentation-related functions, barrier support, moisturization, and anti-aging-oriented claims [7,100,101]. However, these functions should be interpreted through the extract’s chemical profile, intended formulation role, cutaneous exposure, and safety requirements rather than treated as generic skin benefits. The same byproduct-derived fraction may act as an active ingredient, preservative-system-supporting component, fragrance or sensory modifier, structuring material, moisturizer, or upcycled sustainability-oriented ingredient, and each role requires different markers, performance tests, formulation constraints, and safety evidence.

Cosmetic readiness therefore depends not only on antioxidant, antimicrobial, or photoprotective activity, but also on formulation compatibility, color, odor, solubility, pH, viscosity, emulsion or gel stability, photostability, preservative-system compatibility, skin exposure, and local safety assessment [7,76,77]. Phenolic-rich extracts may show strong antioxidant activity but may still alter formulation color, odor, pH, rheology, precipitation behavior, or emulsion stability, particularly when incorporated at high concentrations [76,101]. Pigment- or carotenoid-rich fractions require attention to light, oxygen, pH, and thermal stability, as well as possible staining or color-shift effects [41]. Essential-oil-rich fractions may contribute antimicrobial or aromatic functionality, but they also require control of volatility, odor persistence, oxidation, irritation, sensitization, and potential phototoxicity [102,103,104]. Accordingly, cosmetic-oriented valorization should define the intended function, identify positive and negative quality markers, evaluate formulation behavior and storage stability, and assess cytotoxicity, irritation potential, sensitization risk, phototoxicity where relevant, and preservative-system compatibility under intended-use conditions [7,77,104]. A byproduct-derived extract becomes cosmetic-ready only when its chemical identity, marker reproducibility, formulation performance, skin-safety profile, and sustainability claim are supported by application-relevant evidence, as summarized in Table 5.

### 7.4. Biopesticide and Sustainable Agriculture Applications

Biopesticide and sustainable agriculture applications represent an important but evidence-sensitive pathway for circular valorization [8,105,106]. Extracts or fractions from peels, leaves, pruning waste, pomace, and seed residues may contain defense-related phenolic and flavonoid fractions, terpenoids, essential-oil constituents, alkaloids, glucosinolates, fatty acids, and other specialized metabolites associated with plant defense or stress responses [2,8,105]. Liquid residues such as vegetation waters should be interpreted using aqueous-stream logic, because they are more commonly associated with soluble phenolics, organic acids, process-derived constituents, and residual-load indicators unless additional lipophilic or volatile constituents are supported by profiling. These chemical classes may support antifungal, antibacterial, insecticidal, repellent, nematicidal, herbicidal, elicitor-like, or plant-protective activities, making agri-food byproducts relevant to sustainable crop-protection strategies [8,105,107]. However, biopesticide potential should not be inferred from compound presence or single in vitro inhibition assays alone; activity should be interpreted in relation to target organism, dose–response behavior, extract composition, active markers or fingerprints, assay context, and the intended agricultural use, as summarized in Table 4 and Table 5.

Biopesticide-oriented valorization should therefore be guided by an integrated efficacy–risk–formulation–field relevance framework. Natural origin does not guarantee target selectivity, environmental safety, toxicological acceptability, or stable field performance, particularly when non-target effects, phytotoxicity, environmental persistence, and degradation under realistic conditions are insufficiently evaluated [8,78,79,108]. A crude extract may inhibit a pathogen, insect, nematode, or weed under laboratory conditions but still fail as an agricultural input if it damages the crop, affects beneficial insects or soil biota, loses activity through volatilization, photodegradation, oxidation, rainfall, pH changes, or microbial degradation, or cannot be formulated into a stable and deliverable product [78,79,109]. Candidate extracts should be evaluated for target specificity, crop selectivity, dose–response behavior, mode of action where possible, phytotoxicity, non-target effects, environmental persistence, residue or degradation behavior, formulation stability, delivery performance, and compatibility with integrated pest management and agricultural practices [8,78,79,106]. Chemical profiling can help identify active compounds, marker classes, degradation products, and batch-level fingerprints, but stronger claims require bioassay-guided fractionation, targeted validation, reproducible marker monitoring, formulation testing, greenhouse or field-oriented evaluation, and ecotoxicological assessment when relevant [8,78,110]. For biopesticide—related valorization, the critical question is therefore not only whether a byproduct-derived extract suppresses a target organism, but whether its chemical identity, efficacy, selectivity, formulation behavior, environmental fate, and safety profile support realistic agricultural use, as summarized in Table 5.

### 7.5. Cross-Sector Application Readiness Criteria

Although food, nutraceutical, cosmetic, pharmaceutical, and biopesticide-related pathways differ in evidence thresholds, application readiness can be treated as a cross-sector evidence gate rather than a fixed checklist. At the extract level, core readiness criteria include defined chemical identity, marker- or fingerprint-based standardization, batch reproducibility, documented stability, positive and negative quality markers, contaminant and residual-solvent control, and an acceptable safety profile [2,6,10,45,73]. At the implementation level, readiness also requires solvent and formulation compatibility, process robustness, scale-up feasibility, solvent recovery, cost and techno-economic plausibility, regulatory acceptability, and sustainability or LCA-related evidence [11,80,111]. Without these connected criteria, circular valorization claims may remain limited to proof-of-concept demonstrations of extractability, chemical richness, or preliminary bioactivity rather than evidence of sector-specific usability, as reflected in Table 4 and Table 5.

In this review, application readiness is operationally defined as the extent to which a byproduct-derived extract or fraction can be reproducibly generated, chemically characterized, standardized, stabilized, safely used under the intended exposure context, formulated, and implemented for a defined sector with application-relevant functional evidence. This definition emphasizes that readiness is not a single endpoint but an integrated outcome of matrix selection, green recovery, chemical profiling, profile-linked bioactivity interpretation, quality control, safety assessment, formulation performance, scale-up feasibility, regulatory fit, and sustainability evaluation, as further complemented by the cross-cutting bottlenecks in Table 6. Cross-sector and sector-specific application-readiness criteria are summarized in Table 5. Accordingly, an extract or fraction should not be described as industrially ready solely because it shows efficient laboratory-scale recovery or promising bioactivity. Stronger readiness claims require evidence that the process can be reproduced at pilot or industrially relevant scale, with documented throughput, solvent or water recovery logic, energy demand, process robustness, and preliminary techno-economic or life-cycle considerations where scale-up or sustainability claims are made.

Within this readiness framework, safety assessment is treated as a core evidence gate rather than as a late-stage confirmation step. It should connect source-level hazards with extract-level hazards, including pesticide residues, mycotoxins, heavy metals, microbial contaminants, allergens, antinutritional factors, residual solvents, natural toxicants, process-derived contaminants, and degradation products formed during storage or processing. Because recovery, concentration, drying, stabilization, or formulation can enrich both desired bioactives and undesirable constituents, safety evidence should be interpreted according to concentration factor, exposure route, intended application sector, formulation matrix, and regulatory context.

Regulatory expectations also differ across application sectors and should be interpreted according to product category, exposure route, jurisdiction, and claim type. For food applications, readiness generally depends on food-grade recovery, contaminant and residual-solvent control, permitted ingredient or additive status, sensory suitability, use level, and substantiation of technological or preservation-related function. Nutraceutical and supplement applications require stronger marker standardization, dose or intake rationale, stability, exposure-relevant evidence such as bioaccessibility or bioavailability where relevant, and claim substantiation consistent with the intended use. Cosmetic and dermocosmetic applications require formulation safety, irritation, sensitization, or phototoxicity assessment where relevant, preservation compatibility, topical exposure logic, and claims limited to cosmetic appearance or topical function unless additional route-specific evidence is available. Pharmaceutical discovery or standardized-extract development requires more stringent identity, purity or fractionation status, dose–response evidence, mechanism-oriented validation, cytotoxicity and broader safety assessment, and pharmacological or toxicological evidence before therapeutic claims can be considered. Biopesticide and agricultural applications require target-specific efficacy, dose–response behavior, non-target and phytotoxicity assessment, environmental fate or persistence, formulation durability, and field-relevant performance before plant-protection or agricultural input claims are justified. Thus, application readiness is inherently context-dependent, and permissible claims should be calibrated to the evidence level required for the intended sector.

**Table 5:** Cross-sector and sector-specific application-readiness criteria for agri-food byproduct-derived bioactive extracts.

Readiness Gate and Decision Question	Extract-Level Evidence	Sector or Implementation Evidence	Claim-Limiting Gaps	Representative References
Cross-sector identity and standardization Is the extract/fraction chemically defined, traceable, and reproducible?	Identity/annotation level; marker or fingerprint specifications; batch reproducibility; reporting basis; positive quality markers.	Fit-for-purpose QC; marker ranges; documented feedstock, recovery, profiling, and storage; annotation-confidence and batch-variability criteria.	Yield/TPC/TFC-only claims; unclear markers; weak annotation confidence; batch variability; missing reporting, storage, or fingerprint criteria.	[2,6,73]
Cross-sector stability, safety and negative markers Is the extract stable and safe under intended use?	Stability/degradation indicators; residual-solvent control; contaminant screening; negative markers for pesticides, metals, mycotoxins, microbes, allergens, and undesirable co-extractives.	Storage/formulation stability; exposure-context safety; hazard prioritization; negative-marker thresholds.	Marker loss/degradation; contaminant concentration; residual solvents; unstable pigments/volatiles; missing toxicological, microbiological, allergenicity, or degradation data.	[10,45,112]
Cross-sector implementation, scale-up and sustainability Can it be formulated, scaled, regulated, and implemented?	Solvent compatibility; process robustness; residual moisture; stabilization/carrier needs; solvent, water, and energy demand.	Formulation compatibility; solvent recovery; material/energy balances; pilot or integration logic; TEA plausibility; regulatory pathway; LCA evidence.	Laboratory-only claims; difficult solvent recovery; high drying/energy burden; low throughput; unstable formulations; unclear regulation; unsupported sustainability claims.	[11,80,111]
Food and functional food applications Does it perform in a specific food matrix?	Food-compatible recovery; marker profile; residual solvents/contaminants; sensory-active co-extractives; pigment, redox, microbial, and safety indicators.	In-matrix antioxidant, color-stability, or antimicrobial performance; shelf-life/oxidation/color models; challenge tests; sensory evaluation; matrix interactions; digestion/bioaccessibility.	Off-flavor, bitterness, turbidity, astringency, color shift, pigment instability, macromolecule interactions, altered bioaccessibility, food-safety constraints.	[10,71,72]
Nutraceutical and supplement applications Is it standardized and exposure-relevant?	Marker specifications; batch reproducibility; positive/negative markers; digestive stability; bioaccessibility/bioavailability; safety profile; dose/intake basis.	Dosage/formulation stability; storage stability; exposure-relevant performance; claim-relevant evidence; safety assessment; regulatory categorization.	Profile changes after digestion or microbiota metabolism; weak dose justification; insufficient marker, exposure, bioavailability, safety, or interaction evidence.	[73,74,75]
Cosmetic and dermocosmetic applications Can it function safely in a topical formulation?	Skin-relevant markers; pigment/odor-active constituents; photostability; oxidation/degradation markers; residual solvents/contaminants; irritation/sensitization indicators.	Formulation compatibility; pH, viscosity, color, odor, emulsion/gel stability; preservative compatibility; storage/photostability; cytotoxicity, irritation, sensitization, phototoxicity tests.	Color/odor drift; precipitation; emulsion instability; low solubility; photodegradation; irritation/sensitization risk; unsupported upcycled claims; unclear preservative compatibility.	[7,76,77]
Pharmaceutical discovery or standardized-extract development Is the evidence appropriate for discovery or mechanism validation?	Confirmed or transparent identity/annotation; active-fraction or marker evidence; purity/fractionation status; dose basis; positive/negative markers; safety-relevant impurities or degradation products.	Mechanism-oriented validation; dose–response; orthogonal bioassays; cytotoxicity/safety assessment; target/pathway relevance; stage-appropriate pharmacology/toxicology.	Overclaiming crude in vitro activity; tentative annotations treated as confirmed identities; missing dose–response/mechanism data; insufficient safety, toxicity, pharmacokinetic, or exposure evidence.	[65,94,99]
Biopesticide and sustainable agriculture applications Does it show target-relevant efficacy and field-use readiness?	Active marker/fingerprint evidence; batch reproducibility; volatile/degradation markers; phytotoxicity-relevant constituents; residual solvents/contaminants; storage/application stability.	Target specificity; dose–response; mode-of-action where possible; crop selectivity; non-target assessment; environmental fate; formulation durability; greenhouse/field evaluation; IPM compatibility.	Laboratory inhibition without field relevance; phytotoxicity; non-target effects; environmental persistence; activity loss by volatilization, photodegradation, rainfall, pH, or microbes; formulation failure.	[8,78,79]

Note: Application-readiness criteria are evidence gates rather than final claims. They indicate typical evidence needs, not fixed requirements; final thresholds depend on matrix type, extract profile, intended use, exposure context, jurisdiction, and risk context. References are representative only. Abbreviations: IPM, integrated pest management; LCA, life cycle assessment; QC, quality control; TEA, techno-economic analysis; TFC, total flavonoid content; TPC, total phenolic content.

## 8. Bottlenecks to Translation: Heterogeneity, Standardization, Scale-Up, Stability, Safety, and Sustainability Assessment

Despite the rapid expansion of studies describing agri-food byproducts as reservoirs of high-value bioactive compounds, translation remains constrained by evidence gaps that emerge across the valorization chain, from feedstock selection and recovery to profiling, validation, scale-up, and sustainability assessment. These bottlenecks are not isolated technical problems but connected limitations across the valorization chain: heterogeneous feedstocks can generate inconsistent extract profiles; non-standardized recovery, reporting, and analytical workflows limit cross-study comparability; insufficient chemical identity, annotation-confidence, stability, and safety evidence weakens application claims; and limited formulation, scale-up, techno-economic, regulatory, and sustainability evaluation prevents proof-of-concept studies from becoming feasible circular valorization pathways [2,10,80,111]. Although many studies successfully recover phenolic-, pigment-, lipid-, volatile-, polysaccharide-, or peptide-rich fractions from peels, pomace, seed residues, pruning waste, vegetation waters, and related matrices, the synthesis in this review highlights that fewer studies provide integrated evidence that the recovered materials are reproducible, chemically standardized, stable, safe, scalable, economically plausible, environmentally justified, and suitable for a defined application route. Thus, the translational question is no longer simply whether agri-food residues can yield bioactive extracts, but whether a given matrix–recovery–profile–application combination can satisfy the readiness criteria required for practical circular valorization, as summarized in Table 6.

The following subsections discuss these bottlenecks as interconnected translational gates: feedstock heterogeneity and source-to-extract traceability; recovery-process reporting and chemical-profiling standardization; chemical identity, annotation confidence, and quality-marker definition; bioactivity interpretation and assay relevance; scale-up feasibility and process integration; stability and formulation compatibility; safety and contaminant control; regulatory feasibility; and LCA/TEA-based sustainability assessment. This structure is intended to identify where evidence commonly breaks down and which criteria are needed to move byproduct-derived extracts from laboratory screening toward standardization- and application-readiness-oriented bioactive candidates.

### 8.1. Feedstock Heterogeneity and Batch Variability

Feedstock heterogeneity is one of the most persistent translational bottlenecks because it enters the valorization chain before recovery, profiling, or bioactivity testing begins, as summarized in Table 6. Unlike purified or specification-controlled raw materials, plant-derived agri-food residues are biologically and industrially variable feedstocks generated from different crop types, plant tissues, maturity stages, production environments, and processing streams [113,114]. Their composition may vary according to botanical species, cultivar, tissue type, harvest season, geographic origin, agronomic practice, environmental exposure, and pre-harvest stress conditions, while postharvest handling, industrial processing, drying method, storage duration, and pretreatment can further modify moisture status, dry-matter composition, phytochemical abundance, degradation state, extractability, and bioactivity-related properties [2,113,114,115]. Processing-stream examples, such as olive pomace and olive mill wastewater, further illustrate how residue type and process history can shape phenolic recovery and valorization logic [116]. This variability propagates through the entire recovery–profiling–valorization workflow by affecting extraction yield, solvent demand, compound selectivity, marker content, co-extracted matrix components, contaminant risk, stability, and assay responses [2,10,115]. Therefore, feedstock heterogeneity should be treated as an upstream determinant of extract identity and batch quality rather than as background variation. Thus, feedstock variability should be treated as a standardization gate: marker ranges, fingerprint acceptance criteria, and extract-quality specifications should be calibrated against source metadata rather than assumed to transfer across cultivars, regions, seasons, storage histories, and processing streams.

Managing this variability requires source-to-extract traceability. Studies should document botanical species or crop type, cultivar when available, tissue or residue type, harvest period, geographic or production context, processing route, drying and storage conditions, moisture or dry-matter basis, pretreatment, batch number, batch size, pooling logic, and the basis used for reporting extraction yield and marker content [55,113,114]. Chemical profiling should then be used to establish batch-level fingerprints, marker ranges, acceptance windows, and positive or negative quality indicators related to target composition, degradation, contaminants, or undesirable co-extractives [2,6,45,55]. Importantly, variability should not be interpreted only as a barrier. When properly characterized, it can support feedstock triage by identifying streams that are more suitable for phenolic-rich extracts, lipid-rich fractions, volatile-rich fractions, polysaccharide-rich materials, peptide-rich hydrolysates, or plant-protective and biopesticide-oriented profiles [2,8,81], as reflected in Table 1. In this way, batch variability becomes a decision variable for matrix selection, recovery design, quality-marker definition, and application-route allocation.

### 8.2. Standardization of Recovery and Chemical Profiling

Standardization of recovery and chemical profiling is essential for converting byproduct-derived extracts from isolated experimental outputs into comparable and reproducible materials. Cross-study comparison remains difficult when recovery conditions, reporting bases, extract specifications, analytical workflows, and bioactivity endpoints are described inconsistently. In extraction studies, variables such as solvent type and concentration, temperature, extraction time, pH, solid-to-liquid ratio, particle size, feedstock moisture, pretreatment, equipment configuration, energy input, pressure, and post-extraction processing can all influence the final profile, but they are not always reported with sufficient detail [2,9,36,38]. Reporting bases also require harmonization because extraction yield, marker content, and bioactivity may be expressed on fresh-weight, dry-weight, extract-dry-weight, solvent-volume, or original-feedstock bases, as summarized in Table 6. Similarly, bulk chemical and bioactivity metrics, including total phenolic content, total flavonoid content, antioxidant capacity, inhibition percentage, IC50, EC50, zones of inhibition, and MIC, are useful only when assay conditions, calibration standards, concentration basis, controls, and statistical treatment are clearly specified [18,32,46,62]. Without this level of reporting, differences in yield, chemical composition, or bioactivity may reflect methodological variation rather than true differences in matrix quality or recovery performance, as reflected in Table 4 and Table 6.

A practical standardization framework should include four linked components. First, recovery standardization should document the feedstock basis and process descriptors, including residue type, moisture or dry-matter basis, solvent composition and grade, extraction time, temperature, pH, S:L ratio, particle size, pretreatment, equipment type, energy, pressure, power, or field strength where relevant, and post-extraction concentration, drying, purification, stabilization, or storage conditions [2,9,36]. Second, extract specifications should define solid content or dry matter, residual moisture, extraction yield basis, target compounds or compound classes, major positive markers, diagnostic fingerprints, fingerprint similarity, and acceptable marker ranges [2,6,51,73]. Third, risk-related specifications should include residual solvents, degradation indicators, undesirable co-extractives, pesticide residues, heavy metals, microbial contaminants, mycotoxins, antinutritional factors, allergens, or other negative markers when relevant to the feedstock and intended application [10,11,45,112]. Fourth, analytical standardization should address annotation confidence, matrix effects, calibration strategy, internal standards, quality-control samples, blank correction, method validation or fit-for-purpose validation, data normalization, and reporting of targeted and untargeted features [44,55,117]. Standardization should therefore be viewed not as a formatting exercise, but as the evidence structure that links recovery conditions, extract identity, marker reproducibility, safety-relevant attributes, and application-readiness assessment, as summarized across Table 3, Table 4, Table 5 and Table 6.

### 8.3. Scale-Up Feasibility and Process Integration

Scale-up feasibility should be evaluated as a process-design and evidence-integration problem rather than as a simple extension of laboratory-scale extraction efficiency, as summarized in Table 6. A recovery method that performs well at small scale may become impractical at pilot or industrial scale when solvent throughput, extraction time, energy distribution, heat and mass transfer, pressure requirements, equipment cost, solvent recovery, extract concentration, downstream purification, waste streams, operator safety, and process throughput are considered [9,80,111,118]. Food waste biorefinery literature has repeatedly shown that many valorization pathways remain at laboratory scale, whereas industrial feasibility, feedstock security, techno-economic performance, and environmental assessment are less consistently addressed [80,111,118]. Therefore, scale-up claims should be supported not only by extraction yield or bioactive recovery efficiency, but also by process metrics that indicate whether the recovery route can be operated reproducibly, safely, economically, and with defensible environmental performance. Pilot-scale or demonstration-scale validation is particularly important because laboratory optima may not be preserved when equipment geometry, mixing regime, heat and mass transfer, solvent residence time, feedstock loading, cleaning requirements, and batch size change. Such validation should report run-to-run and batch-to-batch reproducibility, marker retention, solvent recovery and reuse efficiency, energy input per mass of feedstock or extract, throughput, process downtime or cleaning burden, and deviations from laboratory-scale selectivity or extract quality.

Operational robustness differs across green recovery technologies. UAE scale-up requires control of acoustic energy distribution, reactor geometry, mixing, cooling, cavitation intensity, and mass-transfer uniformity; MAE requires uniform heating, pressure control, solvent compatibility, and avoidance of local overheating; SFE requires high-pressure equipment, CO_2_ and co-solvent handling, compression energy, separator design, and reliable solvent recycling; and PLE or subcritical-water processes require stable temperature–pressure control while limiting hydrolysis or thermal degradation of sensitive compounds [2,9,119], as summarized in Table 2. Biological and solvent-engineered processes introduce additional constraints: enzyme- and fermentation-assisted recovery require control of residence time, pH, temperature, substrate moisture, microbial or enzymatic activity, contamination risk, deactivation or stabilization, and downstream purification, whereas DES/NADES-based processes require evaluation of viscosity, dilution effects, mass-transfer limitations, solvent recovery or recyclability, residual solvent control, toxicological uncertainty, safety, and regulatory acceptability [13,15,34,120]. These constraints show that a green technology is not automatically scalable simply because it reduces solvent use or extraction time at laboratory scale.

Process integration with existing food-processing, agricultural, or biorefinery infrastructure is therefore a key determinant of translation. Recovery routes should be assessed according to feedstock logistics, seasonal availability, preprocessing requirements, compatibility with existing unit operations, solvent and water loops, energy sources, waste or effluent management, extract stabilization, and opportunities for cascade or whole-biomass utilization [80,111,118]. Studies claiming industrial relevance should report, where appropriate, material balance, solvent-to-solid ratio, solvent recovery rate, extraction time, energy demand, pressure or power input, throughput, extract concentration, purification load, stabilization or drying requirements, waste-stream generation, and potential integration points, as summarized in Table 6. Life-cycle or techno-economic indicators should also be included when scale-up or sustainability claims are made, because environmental hotspots may arise from electricity use, drying, chemical treatment, solvent production, transport, or downstream concentration rather than from the extraction step alone [111,121,122,123]. Preliminary techno-economic assessment should distinguish capital-related constraints, operating costs, solvent make-up, utility demand, labor and operation requirements, waste treatment, stabilization or drying burden, and downstream purification costs. In parallel, process-efficiency claims should specify whether solvent recovery, water reuse, heat integration, and byproduct logistics are included within the assessment boundary. In this context, the most scalable recovery strategy is not necessarily the one with the highest laboratory yield, but the one that balances target-compound selectivity, operational robustness, solvent and energy efficiency, downstream simplicity, safety, cost, and environmental performance, as summarized in Table 6.

### 8.4. Stability, Safety Assessment, Regulatory Feasibility, and Sustainability Assessment

Stability, safety, regulatory feasibility, and sustainability assessment should be incorporated early in the valorization workflow because they determine whether a chemically active extract can become an application-ready material, as summarized in Table 6. Stability should be viewed as preservation of the intended chemical profile rather than as a generic storage attribute. Many byproduct-derived bioactives are sensitive to oxygen, light, heat, pH, enzymes, microbial activity, solvent environment, and interactions with co-extracted matrix components [2,41,124]. Phenolic-rich extracts may undergo oxidation, polymerization, or loss of redox-active markers; anthocyanins and other pigments may show pH-, light-, oxygen-, or temperature-dependent color and structural changes; carotenoids and tocopherols may isomerize or oxidatively degrade; essential-oil constituents may volatilize or form oxidation products; and peptide-rich fractions may be hydrolyzed or structurally modified during processing, digestion, storage, or formulation [41,97,98,124,125]. Stabilization strategies such as encapsulation, drying, carrier selection, nanoemulsions, microcapsules, and controlled—release systems can improve protection, dispersibility, release behavior, or delivery, but they also introduce additional variables related to carrier identity, loading efficiency, release kinetics, matrix compatibility, cost, scalability, safety assessment, and regulatory acceptability [125,126,127]. Therefore, stability assessment should monitor not only retention of total activity, but also marker preservation, degradation products, physical stability, formulation behavior, and performance under intended-use conditions.

Safety assessment is therefore treated here as a core component of the valorization framework and should cover both the source material and the final recovered extract because valorization may concentrate desirable bioactives together with undesirable constituents, contaminants, or process-derived hazards [10,128,129]. Potential hazards include pesticide residues, mycotoxins, heavy metals, microbial contaminants, biogenic amines, antinutritional factors, allergens, residual solvents, natural toxicants, process-derived contaminants, degradation products formed during storage or processing, and formulation- or processing-derived residues [10,130,131]. Risk profiles should be evaluated according to feedstock origin, agronomic and processing history, storage conditions, recovery method, concentration factor, intended route of use, and exposure scenario. For example, food-processing residues, pruning waste, field residues, liquid effluents, seed residues, and essential-oil-rich fractions may require different safety and regulatory evaluations because they differ in contaminant likelihood, microbial susceptibility, natural toxicants, volatility, solvent compatibility, and end-use exposure [10,129,132]. Regulatory feasibility should therefore be considered as an intended-use-specific evidence requirement, not as a final administrative step, as summarized in Table 6.

Regulatory feasibility is therefore not a universal checklist but a sector- and claim-specific readiness gate. Food, supplement, cosmetic, pharmaceutical, and agricultural or biopesticide uses differ in required safety evidence, quality-control specifications, efficacy substantiation, exposure assumptions, and permissible claim language. Consequently, generic descriptors such as “bioactive,” “natural,” “green,” or “upcycled” should not be extended to health, therapeutic, cosmetic, preservation, or plant-protection claims unless the corresponding sector-specific evidence and regulatory pathway are defined.

Circularity and sustainability claims also require evidence beyond the use of waste-derived feedstock or the label of a green extraction method. Energy demand, solvent production and recovery, water use, drying, transport, preprocessing, concentration or purification, waste-stream management, process integration, life cycle assessment, and techno-economic feasibility should be considered when industrial relevance or environmental benefit is claimed [111,121,122,123]. In some cases, environmental hotspots may arise from electricity consumption, freeze-drying, solvent manufacture, low solvent recovery, chemical pretreatment, or downstream concentration rather than from the extraction step itself [122,123]. Thus, a valorization route should be regarded as circular only when target-compound recovery, extract quality, safety, process feasibility, and environmental performance are jointly supported, as summarized in Table 6. The major translational bottlenecks and corresponding readiness criteria are summarized in Table 6.

**Table 6:** Cross-cutting translational bottlenecks and readiness criteria for circular valorization of agri-food byproduct-derived bioactive extracts.

Evidence Gate and Bottleneck	Why It Limits Translation	Minimum Evidence	Readiness Indicators	Gaps to Avoid	Representative References
Feedstock heterogeneity and source-to-extract traceability	Source variation affects moisture, marker levels, contaminants, extractability, and assay response.	Crop identity; residue type; processing/drying/storage history; moisture or dry-matter basis; batch size and pooling logic.	Batch fingerprints; marker ranges; dry-matter-normalized yield and markers; degradation indicators; traceable source records.	Single-batch claims; vague source; no dry-matter basis; unreported storage; untraceable pooling.	[113,114,115]
Recovery process and reporting standardization	Recovery variables and post-extraction handling can make results non-comparable.	Solvent grade/composition; time; temperature; pH; S:L ratio; particle size; equipment/energy/pressure; pretreatment; post-extraction steps; statistics.	Extract dry matter or solid content; target-fraction definition; unit consistency; solvent/water use; reproducibility; marker acceptance criteria.	Yield- or TPC/TFC-only comparisons; missing variables; inconsistent units; no extract specifications or marker ranges.	[2,36,44]
Chemical identity, annotation confidence, and quality-marker definition	Poor characterization limits comparison, standardization, and biological interpretation.	Fit-for-purpose profiling; confirmed IDs where possible; annotation levels; targeted markers; diagnostic fingerprints; positive/negative quality markers.	Method details; standards/calibration where available; QC samples and blanks; matrix-effect checks; batch classification; marker-panel rationale.	Overstated identities; no annotation level; convenience-only markers; no batch fingerprint; no degradation/contaminant/risk markers.	[6,44,45]
Bioactivity interpretation and assay relevance	Activity may reflect assay format, matrix effects, concentration basis, solubility, turbidity, color, pH, redox interference, or residual solvents.	Profile-linked endpoints; assay context; controls; concentration range; dose–response; normalization; orthogonal assays.	Activity normalized to dry extract, feedstock, or marker content; matrix-compatible controls; replicate structure; statistics; profile linkage; intended-use relevance.	Single in vitro response as proof; TPC/antioxidant-only claims; missing controls or dose–response; no profile link or application-relevant assay.	[61,62,65]
Scale-up feasibility and process integration	Laboratory recovery may fail at scale due to throughput, heat/mass transfer, equipment cost, solvent recovery, purification load, waste, or safety.	Material/energy balances; solvent recovery; throughput; process robustness; equipment needs; feedstock logistics; integration points; drying/stabilization demand.	S:L ratio; batch size or throughput; extraction time; power/pressure input; extract concentration; purification load; waste streams; CAPEX/OPEX proxies.	Laboratory-only industrial claims; no mass balance, solvent recovery, throughput, feedstock logistics, or integration logic.	[80,111,118]
Stability and chemical-profile preservation	Oxygen, light, heat, pH, enzymes, microbes, solvents, and co-extractives can degrade or transform markers.	Stability plan for storage, processing, formulation, and use; marker retention; degradation products; physical stability; defined time/temperature/light/oxygen/moisture.	Marker retention; fingerprint stability; color, odor, or rheology where relevant; degradation markers; water activity or residual moisture; shelf-life logic.	Total activity only; no marker-level stability; no degradation monitoring; unstated storage; one-time composition treated as shelf-life evidence.	[2,41,124]
Stabilization and formulation compatibility	Encapsulation, drying, nanoemulsions, carriers, or release systems may improve delivery but alter release, safety, cost, scalability, sensory quality, and acceptability.	Carrier identity; loading efficiency; encapsulation yield; release behavior; residual moisture; matrix compatibility; storage stability; carrier safety/regulatory rationale.	Particle/droplet size; encapsulation efficiency; release kinetics; dispersibility; formulation stability; sensory/appearance effects; processing compatibility; scale-up logic.	Carriers without safety/regulatory rationale; no release or formulation-stability data; no cost/scale-up discussion; unrealistic protection conditions.	[125,126,127]
Safety assessment and contaminant control	Valorization may concentrate desired bioactives together with pesticides, metals, mycotoxins, microbes, allergens, antinutrients, solvents, or natural toxicants.	Risk-based source/extract assessment; feedstock- and process-specific hazards; contaminant/residual-solvent screening; exposure route; concentration-factor logic.	Pesticides; heavy metals; mycotoxins; microbial load; allergens; antinutritional factors; natural toxicants; residual solvents; process-derived residues.	Safety assumed from natural origin; raw material tested but final extract ignored; no concentration-factor logic; no route-specific exposure assessment.	[10,129,132]
Regulatory feasibility and intended-use fit	Food, supplement, cosmetic, pharmaceutical, and biopesticide/agriculture routes require different solvent, carrier, safety, exposure, efficacy, and residue evidence.	Defined application route; jurisdiction-aware evidence; compliant solvent/carrier/excipient choices; use-specific safety/residue data; evidence-aligned claims.	Residual-solvent and contaminant limits; formulation and exposure route; sector-specific endpoints; non-target or ecotoxicity evidence for biopesticides.	Generic bioactivity claims; direct sector transfer; non-food-grade solvent residues; sector claims without safety, exposure, field, or route-specific evidence.	[8,11,108]
Sustainability, LCA/TEA, and circularity plausibility	Waste-derived feedstocks and green-extraction labels do not guarantee environmental or economic benefit.	Life-cycle boundary or sustainability rationale; cost/TEA indicators; solvent/water/energy demand; transport/preprocessing; solvent recovery; waste management; baseline comparator.	Energy and water use; solvent production/recovery; drying or concentration burden; GHG or endpoint impacts; CAPEX/OPEX proxies; feedstock logistics and yield losses.	Unsupported circularity claims; ignored electricity, freeze-drying, or solvent hotspots; no LCA/TEA boundary; no baseline comparison; no scale-relevant inventory.	[111,121,123]

Note: Bottlenecks are organized as cross-cutting evidence gates that complement the sector-specific readiness criteria in Table 5. Criteria indicate typical evidence needs, not fixed requirements; final thresholds should be refined according to matrix origin, recovery route, extract profile, intended use, exposure context, regulatory jurisdiction, and the level of circularity or sustainability claimed. References are representative only. Abbreviations: CAPEX, capital expenditure; GHG, greenhouse gas; LCA, life cycle assessment; OPEX, operating expenditure; QC, quality control; S:L, solid-to-liquid ratio; TEA, techno-economic analysis; TFC, total flavonoid content; TPC, total phenolic content.

### 8.5. Toward a Translation-Oriented Valorization Framework

The bottlenecks discussed above indicate that agri-food byproduct valorization should be structured as a translation-oriented evidence chain rather than as a sequence of isolated demonstrations, as summarized in Table 6. In this framework, feedstock classification and source-to-extract traceability establish the starting point; matrix-specific green recovery defines target-compound enrichment, co-extractive control, and process feasibility; fit-for-purpose chemical profiling defines extract identity, marker reproducibility, fingerprint comparability, and positive or negative quality markers; profile-linked bioactivity interpretation provides evidence-weighted functional interpretation; and stability, safety, formulation behavior, regulatory feasibility, scale-up potential, techno-economic performance, and sustainability assessment determine whether the recovered material can progress toward sector-specific application readiness [2,6,80,111], as summarized in Table 5 and Table 6.

**Figure 4:**
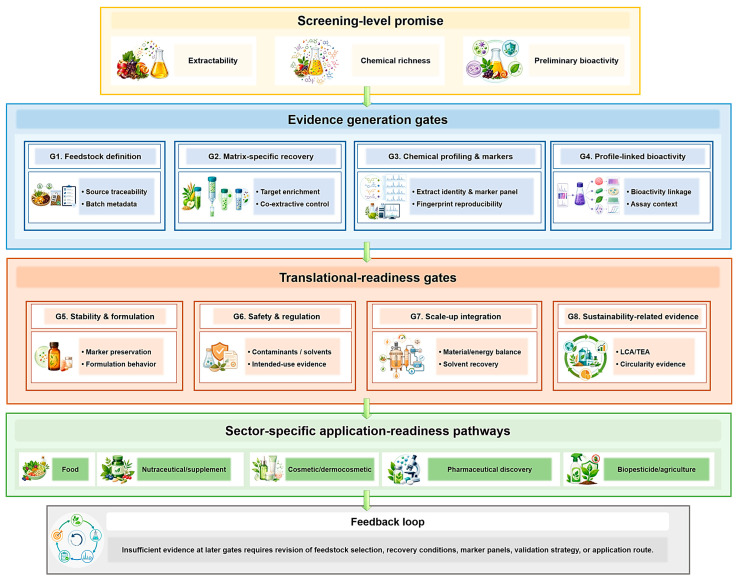
Application-readiness and translational gate model for circular valorization of agri-food byproduct-derived bioactive extracts and fractions.

Each stage should function as a readiness gate that tests whether the evidence generated at one step is sufficient to support the next decision, as illustrated in Figure 4. Feedstock metadata should justify matrix selection and recovery design; recovery conditions should be selected according to target chemistry, selectivity, and downstream compatibility; profiling should define positive and negative quality markers; bioactivity data should be interpreted with chemical profiles, assay context, dose–response behavior, and reproducibility; and implementation claims should be supported by stability, safety, formulation, regulatory, scale-up, techno-economic, and life-cycle evidence [10,111,121], as summarized across Table 4, Table 5 and Table 6. This gate-based logic is especially important for heterogeneous byproduct extracts because chemical activity alone does not ensure reproducibility, safety, formulation compatibility, regulatory acceptability, or sustainability.

Overall, a major barrier to circular valorization is not simply the lack of promising matrices or bioactive compounds, but the incomplete linkage among the evidence types required for translation. Addressing this barrier requires integrated workflows that convert matrix variability into feedstock selection criteria, extraction variables into profile-controlled recovery conditions, chemical profiles into marker specifications, bioactivity results into validated functional evidence, and circularity claims into life-cycle- and techno-economic-supported implementation pathways, as summarized in Figure 4 and Table 4, Table 5 and Table 6. The proposed application-readiness and translational gate model is summarized in Figure 4.

The model organizes translation from screening-level promise, including extractability, chemical richness, and preliminary bioactivity, toward sector-specific application-readiness pathways. Evidence-generation gates include feedstock definition, matrix-specific recovery, chemical profiling and marker specification, and profile-linked bioactivity interpretation. Translational-readiness gates include stability and formulation, safety and regulation, scale-up integration, and sustainability-related evidence, including LCA/TEA and circularity evidence. Sector-specific thresholds differ across food, nutraceutical/supplement, cosmetic/dermocosmetic, pharmaceutical discovery, and biopesticide/agriculture pathways. Feedback loops indicate that insufficient evidence at later gates may require revision of feedstock selection, recovery conditions, marker panels, validation strategy, or application route. The model is intended as an evidence-level and claim-calibration guide rather than a fixed checklist. Abbreviations: LCA, life cycle assessment; TEA, techno-economic analysis.

## 9. Discussion

The central contribution of this review is to reposition agri-food byproduct valorization from a resource-discovery narrative to a translation-oriented evidence-chain problem. The field has clearly demonstrated that residual biomass and liquid or semi-liquid byproduct streams can contain valuable phenolic, lipophilic, volatile, polysaccharide-rich, peptide-rich, and other functional fractions. This progress is important because it has expanded the view of agri-food residues from waste or low-value materials to chemically and functionally relevant feedstocks. However, the practical value of these fractions depends less on chemical richness alone than on whether matrix selection, recovery conditions, chemical profiling, bioactivity interpretation, and application-readiness criteria are connected. In this sense, extractability, total yield, total phenolic content, total flavonoid content, or preliminary antioxidant activity should be interpreted as screening-level promise rather than as evidence of circular valorization success unless extract identity, marker reproducibility, stability, safety, formulation compatibility, scale-up feasibility, and sustainability performance are also addressed [1,2,4,118].

The linkage-oriented framework developed in this review addresses this gap by treating valorization as a connected evidence chain rather than as a linear sequence from residue to extract to application. In this chain, the byproduct matrix defines the starting chemical space, accessibility constraints, and source-related risks; the recovery strategy determines target-compound selectivity, chemical integrity, and co-extracted constituents; chemical profiling establishes extract identity, marker reproducibility, annotation confidence, and standardization potential; bioactivity data become interpretable only when linked to chemical profiles and assay context; and application readiness determines whether the recovered material is suitable for a defined sector-specific pathway. This perspective shifts the central question from whether an agri-food residue contains bioactive compounds to whether a defined matrix–compound–recovery–profile combination can generate a chemically characterized, reproducible, stable, safe, scalable, and application-compatible extract or fraction. The proposed gate-based logic is summarized in Figure 4 and is supported by the evidence-weighting principles outlined in Table 4, Table 5 and Table 6.

A key implication of this framework is that common screening indices should be used for ranking and early comparison, but not for final valorization claims. Extraction yield, total phenolic content, total flavonoid content, antioxidant indices, inhibition percentage, IC50, EC50, zones of inhibition, and MIC values can be informative during early screening, yet they do not define chemical identity, annotation confidence, marker distribution, reproducibility, mechanism, safety, or application relevance. A high-yield extract may contain undesirable co-extractives, residual solvents, unstable compounds, degradation products, excessive pigments, off-flavor precursors, or poorly annotated features, all of which can limit standardization and application readiness, as reflected in Table 5 and Table 6. Conversely, a lower-yield fraction may be more valuable when it has a defined chemical profile, reproducible markers, documented stability, and compatibility with the intended application. Similarly, antioxidant, antimicrobial, anti-inflammatory, photoprotective, enzyme-modulatory, cell-based, and plant-protective responses should be treated as application-relevant evidence only when they are interpreted with chemical-profile information, assay conditions, controls, dose–response behavior, safety considerations, and realistic use scenarios. This distinction is consistent with the interpretation framework in Table 4, where profile–bioactivity integration is treated as hypothesis generation and evidence prioritization rather than direct proof of causality [2,18,32,46].

Chemical profiling therefore acts as the boundary between exploratory screening and credible standardization. HPLC-DAD, LC-MS/MS, LC-HRMS, GC-MS/GC-FID, NMR, FTIR/NIR/Raman, and chemometric or metabolomics-based workflows provide complementary evidence for compound identification, metabolite annotation, marker quantification, fingerprint comparison, batch classification, and profile–bioactivity interpretation, as summarized in Table 3. However, the value of these platforms depends not only on analytical resolution but also on interpretation quality. Tentative metabolite annotations should not be treated as confirmed compound identities unless supported by authentic standards, matched retention behavior, MS/MS evidence, or other sufficient structural evidence. Likewise, fingerprint-based discrimination should not be overinterpreted without appropriate validation, batch structure, preprocessing transparency, and model checking. For application-oriented valorization, profiling should also move beyond positive markers associated with desirable bioactives and include negative markers associated with contaminants, degradation products, residual solvents, unstable constituents, allergenicity- or toxicity-relevant features, and other process-related risks. This dual use of positive and negative markers is essential for transforming chemically complex extracts into materials that can be compared, standardized, and evaluated for readiness [6,44,45,52].

Application readiness provides a second interpretive filter by distinguishing promising extracts from merely active extracts. Food, nutraceutical/supplement, cosmetic/dermocosmetic, pharmaceutical discovery, and biopesticide/agriculture pathways differ in evidence thresholds, formulation constraints, regulatory expectations, exposure scenarios, and performance requirements. A phenolic-rich extract with antioxidant activity may be relevant for food preservation, but it may be insufficient for nutraceutical standardization without reproducible marker content, stability, exposure-relevant evidence, and safety documentation. A pigment-rich fraction may be attractive as a natural colorant but fail because of pH-, light-, oxygen-, or heat-dependent instability. An essential-oil-rich fraction may provide antimicrobial or aromatic functionality but raise concerns related to volatility, oxidation, irritation, sensitization, odor persistence, or formulation compatibility. A crude extract with in vitro biopesticidal activity may fail under greenhouse or field conditions because of phytotoxicity, non-target effects, environmental persistence, photodegradation, rainfall sensitivity, microbial degradation, or delivery limitations. Therefore, application-route selection should be guided by chemical profile, marker reproducibility, stability, safety profile, formulation behavior, scale-up feasibility, regulatory feasibility, and sustainability assessment rather than inferred from preliminary bioactivity alone, as summarized in Table 5 [2,8,10,11].

The framework also clarifies why translation remains difficult despite the abundance of promising matrices and bioactive compounds. A major barrier is not simply the lack of extractable phytochemicals, but the incomplete linkage among the evidence types required for translation. Feedstock heterogeneity must be converted into source-to-extract traceability; extraction variables must be converted into profile-controlled recovery conditions; chemical profiles must be converted into marker specifications and quality-control criteria; bioactivity results must be converted into validated functional evidence; and circularity claims must be supported by scale-up, techno-economic, and life-cycle evidence. This is particularly important for agri-food byproducts because cultivar, tissue type, maturity, storage, processing history, pretreatment, and residual matrix composition can reshape extractability, marker abundance, degradation status, contaminant likelihood, assay response, and downstream compatibility. In this sense, the most promising agri-food byproduct resources are not necessarily those with the highest extraction yield or strongest preliminary activity, but those for which chemical identity, reproducible quality, demonstrated stability, safety, process feasibility, and application-specific functionality can be supported by connected evidence, as summarized in Table 6 [10,80,111,121].

This review should also be interpreted within the limits of its design. Because it was developed as a critical narrative review rather than a systematic review, quantitative meta-analysis, or bibliometric study, the proposed framework should not be read as a numerical ranking of byproduct matrices, recovery technologies, profiling platforms, or application sectors. Instead, it is intended as an evidence-organization and claim-calibration tool. The framework does not imply that every exploratory study must satisfy all translational gates. Early-stage studies may appropriately provide screening-level evidence, especially when their purpose is to identify promising matrices, recovery windows, candidate markers, or preliminary bioactivities. However, stronger claims—such as standardized extract development, application readiness, circular implementation, or sector-specific usability—require progressively stronger evidence. Aligning claim strength with evidence level is therefore essential for avoiding overextension from chemical richness or crude activity to unsupported application or circularity claims.

Future studies would be more informative if they adopted this evidence-chain logic at the design stage. Depending on the intended claim strength, studies should define feedstock source and processing history, select recovery methods according to target chemistry and intended use, report recovery and profiling conditions transparently, distinguish confirmed identifications from tentative annotations, link bioactivity data to chemical markers or fingerprints, evaluate stability and safety under intended-use conditions, and assess formulation, scale-up, regulatory, techno-economic, and environmental constraints when application or sustainability claims are made. Such reporting would make it possible to distinguish screening-level evidence from profile-supported evidence, candidate-validated evidence, application-oriented evidence, and translation-oriented evidence, including scale-up, regulatory, techno-economic, and life-cycle support. This evidence-aware reporting would improve comparability across matrices, technologies, and sectors while helping identify genuinely translatable valorization strategies rather than proof-of-concept demonstrations.

Overall, the proposed framework supports a more cautious and operational interpretation of circular valorization. Agri-food byproducts should not be valued solely because they contain bioactive compounds, nor should green recovery technologies be considered successful solely because they increase extraction yield or reduce solvent use. Practical circular valorization requires that matrix variability, recovery selectivity, chemical profiling, bioactivity interpretation, application readiness, and sustainability evidence be linked. By converting matrix variability into feedstock selection criteria, extraction variables into profile-controlled recovery conditions, chemical profiles into marker specifications, bioactivity results into validated functional evidence, and circularity claims into life-cycle- and techno-economic-supported implementation pathways, the field can move toward standardized, application—readiness-oriented bioactive candidates from agri-food byproducts.

## 10. Conclusions

Agri-food byproducts are promising renewable feedstocks for high-value bioactive compounds, but their circular valorization requires more than recovery yield, total phenolic content, or preliminary in vitro activity. This review highlights that practical valorization depends on a connected evidence chain linking feedstock characteristics, green recovery conditions, chemical profiling, bioactivity interpretation, quality control, and sector-specific application readiness. In this context, chemical profiling is not a final descriptive step but an interpretive and standardization tool that connects recovery selectivity with extract identity, marker reproducibility, stability, safety, and application-relevant performance.

The proposed framework should therefore be used as a claim-calibration tool rather than as a fixed checklist. Screening-level evidence may be sufficient for early prioritization, but stronger application-readiness claims require progressively stronger support, including compound-level or fingerprint-level characterization, reproducible marker ranges, safety assessment, stability data, formulation compatibility, scale-up feasibility, techno-economic or life-cycle evidence where relevant, and sector-specific regulatory alignment. The most promising byproduct resources are not necessarily those with the highest extraction yield or strongest preliminary assay response, but those for which matrix variability, recovery selectivity, chemical identity, bioactivity evidence, safety, process feasibility, and intended application can be connected in a reproducible and transparent manner.

Several challenges remain before byproduct-derived bioactive extracts can be translated more broadly into food, nutraceutical, cosmetic, pharmaceutical, biopesticide, or agricultural applications. These include raw-material heterogeneity, insufficient source-to-extract traceability, limited standardization of marker and fingerprint criteria, incomplete safety and contaminant assessment, limited pilot-scale validation, uncertain solvent and energy balances, and sector-specific regulatory constraints. Future studies should therefore move beyond isolated extraction or activity endpoints and report integrated evidence on source variability, recovery conditions, chemical profiles, safety, stability, formulation behavior, scale-up performance, and permissible application claims. Such an integrated approach can support the development of standardized, evidence-supported, and application-readiness-oriented bioactive candidates from agri-food byproducts.

## Figures and Tables

**Table 1:** Representative agri-food byproduct matrices, major bioactive compound classes, and valorization considerations.

Byproduct Matrix or Source Stream	Dominant Recoverable Fractions	Recovery and Profiling Priorities	Potential Valorization Pathways	Application-Readiness Considerations	Representative References
Fruit and vegetable peels (citrus; apple; mango; pomegranate; onion; tomato)	Phenolics/flavonoids; citrus PMFs/limonoids; carotenoids/pigments; volatile terpenoids/essential oils; pectin/fiber/waxes.	Phenolics: hydroethanolic extraction, UAE/MAE/PLE. Volatiles: hydrodistillation, solvent-free MAE, or SFE. Pectin/fiber: acid, EAE, or green solvents. Profiling: LC-DAD/LC-MS; GC-MS/GC-FID.	Food preservation/color; pectin/fiber ingredients; functional food/nutraceutical; cosmetics; aroma/essential-oil fractions; plant-protective or biopesticide-oriented profiles.	Pesticide residues, waxes, sugars/acids, bitterness/astringency, odor-active volatiles, pigment instability; sensory quality, safety, marker reproducibility, and standardization.	[2,3,19,20,21]
Pomace and pressing residues (grape; apple; tomato; olive; juice, puree, wine, or oil residues)	Mixed skins/pulp/seeds/stems/fibers; phenolic acids, flavan-3-ols, anthocyanins, proanthocyanidins, stilbenes; carotenoids/pigments; pectin/fiber; residual oils/fatty acids.	Define anatomical fraction first. Match polar, pigment, lipid, pectin/fiber, or mixed-profile recovery to target. Profiling: LC-MS, LC-DAD, fingerprints; add lipid/fatty-acid profiling when seeds are relevant. Report dry-matter, yield, and concentration bases.	Functional-food ingredients; antioxidant/colorant candidates; nutraceutical/cosmetic candidates; circular biorefinery fractions; profile-guided discovery materials.	Heterogeneity and tissue partitioning; free/conjugated/polymerized/bound forms; co-extracted sugars, acids, proteins, pigments, lipids, fibers; turbidity, storage, microbial, sensory/formulation issues.	[2,4,22,23,24]
Seed residues, hulls, shells, and bran fractions (cereal/rice bran; oilseed meals; nut shells; legume and fruit seed residues)	Fibrous tissues: bound hydroxycinnamates, ferulic acid, tannins, lignans, fiber, polysaccharides, lignocellulose. Kernels/meals: oils, fatty acids, tocopherols, phytosterols, proteins/peptides.	Separate lipid-rich and fibrous fractions. Use milling, defatting, alkaline treatment, EAE, fermentation, UAE/MAE, PEF, or solvent engineering. Monitor free/released/bound phenolics, lipid oxidation, tocopherols, hydrolysis degree, MW/peptide quality.	Phenolic antioxidant fractions; lipid-oriented fractions; peptide hydrolysates; fiber/polysaccharides; nutraceutical/supplement candidates; plant-protective fractions.	Limited accessibility; oxidation; antinutritional or undesirable co-extractives; excessive hydrolysis; bitterness, allergenicity, sensory constraints; unit basis and marker preservation.	[2,3,25,26]
Pruning waste, leaves, and stems (orchards, vineyards, olive groves, and herbaceous systems)	Defense phenolics/flavonoids; olive sector secoiridoids, including oleuropein- and hydroxytyrosol-related compounds; terpenoids/triterpenes; lignans/alkaloids; selected volatiles; lignocellulose.	Maintain source-to-extract traceability. Control cultivar/season/agronomy, tissue age, handling, drying, and particle size. Select polar, terpenoid, or secoiridoid-oriented recovery. Profiling: LC-MS/LC-DAD, GC-MS, or fingerprints.	Food, cosmetic, and nutraceutical candidates; plant-protective or biopesticide-oriented profiles; agricultural valorization; phenolic-, terpenoid-, or secoiridoid-enriched fractions.	Field variability; cultivar/season effects; contaminants; post-collection instability; drying/storage losses; lignocellulosic barriers; variable marker preservation; dispersed-biomass scale-up.	[2,27,28,29]
Vegetation waters and liquid or semi-liquid residues (olive mill wastewater; vegetation waters; aqueous effluents)	Soluble aqueous phenolics, especially hydroxytyrosol-, tyrosol-, and secoiridoid-derived compounds; organic acids; colloidal/process-derived constituents; microbial and organic-load indicators.	Use aqueous-effluent logic. Clarify, centrifuge/filter, use membranes, resin adsorption/concentration, liquid–liquid or solid–liquid extraction, and treatment/biorefinery integration. Profiling should track phenolics, detoxification, residual load, and microbial risk.	Phenolic recovery; water-treatment-linked valorization; food/cosmetic/pharmaceutical fractions only after safety/stability evidence; agricultural reuse after detoxification and regulatory alignment; integrated biorefinery streams.	High water content and COD/BOD; microbial risk; colloids; storage instability; concentration/solvent-recovery burden; environmental toxicity; detoxification and safe reuse/disposal needs.	[2,29,30,31]

Note: Matrix–compound assignments indicate common tendencies and recovery hypotheses rather than fixed classifications; final priorities depend on matrix origin, cultivar or species, processing and storage history, pretreatment, target fraction, extract identity and quality attributes, and intended application. The rightmost column lists representative references only. Abbreviations: BOD, biochemical oxygen demand; COD, chemical oxygen demand; DAD, diode array detection; EAE, enzyme-assisted extraction; GC-FID, gas chromatography–flame ionization detection; GC-MS, gas chromatography–mass spectrometry; LC-DAD, liquid chromatography with diode array detection; LC-MS, liquid chromatography–mass spectrometry; MAE, microwave-assisted extraction; MW, molecular weight; PEF, pulsed electric field; PLE, pressurized liquid extraction; PMFs, polymethoxylated flavones; SFE, supercritical fluid extraction; UAE, ultrasound-assisted extraction.

**Table 2:** Green recovery strategies for bioactive compounds from agri-food byproducts: mechanisms, suitable targets, advantages, limitations, and scale-up considerations.

Recovery Strategy	Mechanistic and Process Basis	Suitable Target Fractions and Matrices	Main Advantages	Main Limitations and Quality Risks	Scale-Up and Application-Readiness Considerations	Representative References
Conventional and food-grade solvent extraction (reference baseline)	Solvent diffusion/partitioning using water, ethanol, hydroethanolic, or GRAS solvents; maceration, SLE, reflux, Soxhlet, hydrodistillation.	Baseline for phenolics, pigments, pectin/fiber, semi-polar fractions, and essential oils from peels, pomace, bran, and pruning residues.	Simple, accessible baseline for solvent effects, matrix accessibility, kinetics, and emerging technologies.	High solvent, time, and energy demand; heat/light/oxygen damage; co-extraction; volatile loss; residual-solvent concerns.	Report solvent grade/composition, S:L ratio, time/temp., pretreatment, yield basis, solvent recovery, and profiling data.	[2,4,36]
Ultrasound-assisted extraction (UAE)	Acoustic cavitation and microstreaming enhance matrix disruption, solvent penetration, and mass transfer.	Phenolics, flavonoids, anthocyanins, pigments, pectins, and selected volatile-associated fractions from peels, pomace, leaves, pruning residues, bran-like matrices.	Shorter time; lower solvent use; food-grade solvent compatible; mild/moderate-temperature recovery.	Excess power/time or poor cooling may cause heating, oxidation, emulsification, clarification difficulty, or marker loss.	Control energy density, reactor geometry, mixing, cooling, particle size, solvent system, and temp. profile; report marker preservation.	[2,3,22]
Microwave-assisted extraction (MAE)	Dielectric heating accelerates cell rupture, internal pressure development, and compound release.	Phenolics, flavonoids, anthocyanins, pigments, pectin-rich fractions, microwave-responsive matrices, and selected volatiles under controlled conditions.	Rapid and solvent-saving; efficient when solvent polarity and water content match the matrix.	Non-uniform heating, pressure buildup, excess power, or poor cooling may degrade thermolabile markers, promote isomerization, or cause volatile loss.	Control penetration depth, field distribution, pressure, temp. history, solvent compatibility, loading, and heat removal; report power/time/temp.	[2,3,22]
Enzyme-assisted extraction (EAE)	Enzymes weaken cell-wall, pectin, protein, glycosidic, or ester-linked barriers under mild conditions.	Bound phenolics, phenolic acids, peptides, oligosaccharides, pectin, polysaccharides, and fiber-associated fractions from bran, hulls, peels, pomace, and seeds.	Mild and selective for protected compounds; aqueous/food-grade compatible.	Specificity, cost, dose, pH, temp., time, residual activity, and matrix variability can shift profiles or generate mixed products.	Standardize enzyme type/dose, moisture, time, pH/temperature, inactivation, clarification, and marker monitoring; distinguish release from transformation.	[2,9,16]
Fermentation-assisted recovery	Microbial enzymes disrupt matrices and biotransform glycosylated, polymerized, protein-bound, or cell-wall-associated metabolites.	Bound phenolics, peptides, oligosaccharides, polysaccharide-derived fractions, and transformed metabolites from bran, pomace, seed meals, peels, protected residues.	Releases bound compounds; generates metabolites; may reduce antinutrients; can improve digestibility and functional/sensory profiles.	Strain variability, contamination, off-flavors, uncontrolled biotransformation, residual activity, and complex mixtures complicate standardization.	Define strain/inoculum; control pH, moisture, temperature, and time; verify reproducibility, microbial safety, stabilization, and profiling.	[13,15,16]
Supercritical fluid extraction (SFE)	Supercritical CO_2_ provides tunable low-polarity solvent density; pressure, temperature, and co-solvents extend the polarity range.	Carotenoids, tocopherols, fatty acids, phytosterols, waxes, terpenoids, and essential-oil constituents from seeds, pomace, peels, leaves, aromatic residues.	Low residual-solvent burden; selective lipophilic/volatile recovery; useful for oxygen-sensitive targets; CO_2_ recyclable.	Limited polarity without co-solvent; high-pressure equipment, compression energy, co-solvent handling/removal, and cost constraints.	Assess pressure, temperature, CO_2_ flow, co-solvent ratio, separator design, recycling, throughput, fractionation, marker stability, and LCA/TEA.	[2,22,35]
Pressurized liquid/pressurized hot-water extraction (PLE/PHWE)	Elevated temperature/pressure maintain solvents as liquids and enhance penetration, diffusivity, solubility, and desorption; PHWE tunes water polarity.	Phenolics, flavonoids, anthocyanins, pectin-associated fractions, polysaccharides, peptides, organic acids, and bound fractions from peels, pomace, bran, winery residues, coffee waste, aqueous matrices.	Efficient mass transfer; shorter processing; water/ethanol compatible; tunable solvent properties; rapid or automated operation.	High temperature or prolonged extraction may cause hydrolysis, oxidation, isomerization, Maillard-type changes, thermolabile loss, or co-extraction.	Control temperature–pressure profile, residence time, solvent recycling, corrosion risk, concentration, energy/water demand, throughput, degradation markers, and purification/drying.	[2,37,38]
Pulsed electric field-assisted pretreatment (PEF)	Short electric pulses induce electroporation and cell permeabilization, improving mass transfer before or during extraction.	Wet, pigment-rich, aromatic, or oil-bearing matrices; peels, pomace, leaves, and liquid/semi-liquid streams before extraction, pressing, or diffusion recovery.	Non-thermal/mild pretreatment; may reduce time, solvent demand, or temperature when integrated with compatible extraction.	Performance depends on conductivity, moisture, field strength, pulse number, energy input, electrode design, and temperature rise; dry matrices require conditioning.	Optimize chamber/electrode design, field strength, pulse number, energy input, conductivity, temperature, safety, cleaning, and downstream integration; report PEF as pretreatment when appropriate.	[2,9,39]
Deep eutectic and natural deep eutectic solvent extraction (DES/NADES)	Hydrogen-bond donor/acceptor networks tune polarity, viscosity, hydrogen bonding, and solvent–solute interactions.	Phenolics, flavonoids, anthocyanins, pectins, polysaccharides, and polar/semi-polar bioactives from food/agro-industrial residues; often paired with UAE or MAE.	Low volatility; tunable solubility/stability; compatible with green extraction or direct-use concepts when acceptable for the intended application.	High viscosity and dilution needs; slow mass transfer; difficult recovery; residual-solvent control; toxicological/ecotoxicological and regulatory uncertainty; analytical matrix effects.	Assess preparation, water content, recyclability, purification or direct use, viscosity, component safety, residual limits, regulatory acceptance, and marker-quantification compatibility.	[14,34,40]
Volatile-preserving and solvent-free recovery (hydrodistillation, steam distillation, solvent-free MAE)	Volatilization, vapor transfer, condensation, or microwave-driven release of essential oils and aroma-active fractions with little or no organic solvent.	Essential oils, volatile terpenoids, aroma-active aldehydes, alcohols, esters, and semi-volatiles from citrus peels, aromatic leaves, pruning residues, herbs, and aroma-rich byproducts.	Avoids/reduces organic solvents; yields food-, cosmetic-, antimicrobial-, or plant-protective volatile fractions when thermal exposure is controlled.	Thermal exposure, hydrolysis, oxidation, isomerization, emulsions, volatile loss, water/energy demand, and aroma artifacts may alter extract identity and sensory quality.	Report distillation/microwave conditions, oxygen/light exposure, water/energy use, yield basis, storage, and GC-MS/GC-FID data with retention indices; check irritation, sensitization, or phototoxicity when relevant.	[2,22,33]

Note: Conventional and food-grade solvent extraction is included as a reference baseline. Recovery-strategy assignments indicate typical starting points, not fixed prescriptions. Final selection should consider matrix structure, target chemistry, extract identity and quality attributes, safety, downstream compatibility, intended application, scale-up feasibility, and sustainability-related evidence. References are representative only. Abbreviations: DES, deep eutectic solvents; EAE, enzyme-assisted extraction; GC-FID, gas chromatography–flame ionization detection; GC-MS, gas chromatography–mass spectrometry; GRAS, generally recognized as safe; LCA, life cycle assessment; MAE, microwave-assisted extraction; NADES, natural deep eutectic solvents; PEF, pulsed electric field; PHWE, pressurized hot-water extraction; PLE, pressurized liquid extraction; SFE, supercritical fluid extraction; S:L, solid-to-liquid ratio; SLE, solid–liquid extraction; TEA, techno-economic analysis; UAE, ultrasound-assisted extraction.

**Table 3:** Chemical profiling platforms for agri-food byproduct-derived bioactive compounds: analytical outputs, suitable targets, strengths, limitations, and standardization value.

Profiling Platform or Analytical Approach	Analytical Outputs and Suitable Targets	Key Strengths	Main Limitations and Quality Risks	Standardization and Application-Readiness Value	Representative References
HPLC-DAD/UHPLC-DAD	Known UV-Vis-absorbing markers; retention time, UV-Vis spectra, peak areas, targeted/semi-targeted quantification, and chromatographic fingerprints. Targets: phenolic acids, flavonoids, anthocyanins, stilbenes, tannins, secoiridoids, carotenoids, and degradation markers.	Accessible first-line platform for routine recovery-method comparison, batch monitoring, and stability tracking when standards are available.	Limited structural confirmation; co-elution, isomers, glycosylated/acylated derivatives, polymeric tannins, degradation products, and unavailable standards can lower confidence.	Known-marker quantification and QC; peak-pattern comparison; batch reproducibility checks; preliminary degradation monitoring.	[2,4,24]
Targeted LC-MS/MS (MRM/PRM)	Predefined marker control; precursor/product ion transitions, MS/MS evidence, low-level detection, and calibration/internal-standard-supported quantification. Targets: phenolic, flavonoid, secoiridoid, carotenoid-derivative, lipid-associated, degradation, or negative markers.	High sensitivity and selectivity for predefined markers; useful for validated ranges, acceptance windows, and batch-level standardization.	Requires reference compounds or well-defined transitions; matrix effects, ion suppression, clean-up, calibration design, and validation determine reliability.	Quantitative marker specifications; targeted positive/negative markers; selected degradation markers; standardized release criteria.	[2,24,44]
LC-HRMS and untargeted metabolomics	Broad feature discovery; accurate mass, isotope patterns, adducts, neutral losses, MS/MS spectra, feature tables, tentative annotations, and discriminant features. Targets: complex phenolics, glycosides, tannins, secoiridoids, lipophilic/degradation-related metabolites, and unknown markers.	Broad chemical coverage; useful for discovery, cultivar/process comparison, marker prioritization, and profile-bioactivity hypothesis generation.	Annotations may remain tentative; adducts, in-source fragments, isomers, co-elution, matrix effects, batch effects, and overfitting can weaken interpretation.	Annotation-confidence reporting; discriminant fingerprinting; candidate-marker selection; batch classification; validation planning.	[44,52,53]
GC-MS/GC-FID	Volatile/semi-volatile profiling; mass spectra, retention indices, volatile fingerprints, relative abundance profiles, and GC-FID-supported quantification. Targets: essential-oil constituents, aroma-active compounds, terpenoids, aldehydes, alcohols, esters, FAMEs, and derivatized small metabolites.	Well suited to volatile and aroma-rich fractions; links volatile fingerprints to sensory, antimicrobial, antifungal, or plant-protective relevance.	Library matching alone is insufficient; co-elution, isomeric terpenoids, thermal artifacts, oxidation products, derivatization bias, and RI misuse can cause over-assignment.	RI-supported volatile fingerprints; odor/stability markers; QC for essential-oil-rich or volatile-preserving recovery routes.	[47,48,49]
LC-DAD/LC-MS lipid-oriented profiling	Lipophilic-marker profiling; UV-Vis spectra, retention behavior, targeted LC/MS evidence, lipid-class patterns, and oxidation-related markers. Targets: carotenoids, tocopherols, phytosterols, fatty acids, wax-related constituents, lipophilic pigments, and oxidation-sensitive markers.	More appropriate than routine GC-MS for less volatile or thermally sensitive lipophiles unless validated derivatization is used; supports stability tracking.	Light, oxygen, and heat sensitivity; isomerization, oxidation, low solubility, and lipid co-extractives can affect recovery and quantification.	Lipophilic marker specifications; stability tracking; oxidation-marker monitoring; assessment of SFE or lipophilic-solvent recovery outputs.	[2,6,41]
NMR spectroscopy	Global spectral fingerprints; structural-class evidence; relative/absolute quantification in selected workflows; mixture-level metabolite patterns. Targets: major metabolites and class-level signatures in complex extracts, including sugars, organic acids, phenolics, lipids, and purified/semi-purified fractions.	Highly reproducible and non-destructive; minimal sample preparation; useful for holistic comparison, authenticity/origin discrimination, and batch-level fingerprinting.	Lower sensitivity than MS; peak overlap and complex matrices can limit minor-compound detection and confident assignment without complementary data.	Orthogonal profile comparison; batch classification; stability assessment; confirmation of major compositional shifts.	[6,54,55]
FTIR/NIR/Raman fingerprinting	Rapid vibrational fingerprints; functional-group spectra, class-level chemical signatures, and multivariate spectral variables. Targets: polysaccharide-, fiber-, protein-, lipid-, or phenolic-rich extracts, powders, and fractions requiring rapid screening or batch comparison.	Fast, low-solvent or non-destructive screening; suitable for routine QC, classification, and monitoring of processing or stability-related changes.	Limited compound-level identification; preprocessing choices, spectral overlap, water content, and model overfitting can distort interpretation.	Fingerprint similarity; batch classification; process monitoring; quality-marker selection when coupled with validated chemometrics.	[6,50,51]
Macromolecular and sequence-oriented profiling	Polymer/sequence-level attributes; molecular-weight distribution, SEC/GPC profiles, monosaccharide composition, degree of hydrolysis, and amino acid or peptide profiles. Targets: pectin, dietary fiber, polysaccharides, proteins, peptide hydrolysates, and cell-wall-associated fractions from bran, peels, pomace, hulls, or seed residues.	Captures attributes not visible in small-molecule profiling, including polymer size, hydrolysis state, peptide distribution, and structural/fraction quality.	Requires complementary methods; purification, incomplete hydrolysis, matrix salts, protein/lipid co-extractives, and method-dependent calibration can affect results.	Macromolecular specifications; hydrolysis indicators; peptide/fiber markers; functional attributes relevant to digestion, texture, or formulation.	[2,25,26]
Chemometrics and multi-platform marker panels	Integrated profile interpretation; PCA, HCA, PLS/OPLS, regression/classification outputs, targeted markers, fingerprints, and positive/negative marker panels. Targets: complex extracts requiring batch classification, origin/process discrimination, profile-bioactivity linkage, candidate marker prioritization, or readiness QC.	Integrates targeted and untargeted evidence; helps convert chemical profiles into marker panels, acceptance windows, and evidence-weighted interpretation.	Model validity depends on sample size, preprocessing, batch design, validation, and confirmation; overfitting can create non-biological conclusions.	Extract identity; marker reproducibility; positive/negative QC markers; contamination/degradation indicators; application-readiness assessment.	[6,45,51]
Negative-marker and safety-oriented profiling	Risk-marker control; targeted contaminant assays, residual-solvent checks, pesticide, mycotoxin, heavy-metal, allergen/antinutritional-factor, degradation-marker, and microbial-risk indicators. Targets: application-directed extracts requiring safety, contaminant control, degradation-state, or residual-process-risk documentation.	Complements desirable-bioactive profiling; supports risk-based QC, contaminant control, and sector-specific readiness claims.	Hazard selection must be matrix- and use-specific; absence of screening for relevant risks can create false confidence.	Negative quality markers; contaminant limits; degradation thresholds; residual-risk documentation; evidence for application-oriented valorization.	[10,44,45]

Note: Profiling-platform assignments indicate fit-for-purpose starting points rather than fixed analytical prescriptions; final selection should be refined according to target chemistry, matrix complexity, recovery strategy, available standards, annotation confidence, intended application, and required positive or negative quality markers. The rightmost column lists representative references only. Abbreviations: DAD, diode array detection; FAMEs, fatty acid methyl esters; FID, flame ionization detection; FTIR, Fourier-transform infrared spectroscopy; GC, gas chromatography; GC-FID, gas chromatography–flame ionization detection; GC-MS, gas chromatography–mass spectrometry; GPC, gel permeation chromatography; HCA, hierarchical cluster analysis; HPLC, high-performance liquid chromatography; HPLC-DAD, high-performance liquid chromatography with diode array detection; HRMS, high-resolution mass spectrometry; LC, liquid chromatography; LC-DAD, liquid chromatography with diode array detection; LC-HRMS, liquid chromatography–high-resolution mass spectrometry; LC-MS, liquid chromatography–mass spectrometry; LC-MS/MS, liquid chromatography–tandem mass spectrometry; MRM, multiple reaction monitoring; MS, mass spectrometry; MS/MS, tandem mass spectrometry; NIR, near-infrared spectroscopy; NMR, nuclear magnetic resonance; OPLS, orthogonal projections to latent structures; PCA, principal component analysis; PLS, partial least squares; PRM, parallel reaction monitoring; QC, quality control; RI, retention index; SEC, size-exclusion chromatography; SFE, supercritical fluid extraction; UHPLC, ultra-high-performance liquid chromatography; UHPLC-DAD, ultra-high-performance liquid chromatography with diode array detection; UV–Vis, ultraviolet–visible spectroscopy.

## Data Availability

This review synthesizes findings from existing literature, with all sources referenced within the article. No new data were generated or analyzed in this study.
